# Genome-Wide Characterization, Stress-Responsive Expression, and QTLome Integration of the DUF1645 Gene Family in Rice (*Oryza sativa* L.)

**DOI:** 10.3390/genes17091044

**Published:** 2026-08-29

**Authors:** Peipei Su, Zhiqun Que, Xin Song, Gehong Wang

**Affiliations:** School of Life Sciences and Environmental Resources, Yichun University, Yichun 336000, China

**Keywords:** rice, DUF1645 family, QTLome, abiotic stress, expression pattern

## Abstract

**Background**: Domain of Unknown Function 1645 (DUF1645) is a conserved but poorly characterized plant gene family whose evolutionary history and roles in stress adaptation remain unclear. We performed an integrated genomic, evolutionary, transcriptomic, and Quantitative Trait Locus (QTL) characterization of the DUF1645 family in rice (*Oryza sativa*). **Methods and Results**: We identified 14 intronless, non-redundant OsDUF1645 genes distributed across eight chromosomes. Phylogenetic and collinearity analyses suggested that family expansion within Poaceae involved ancestral segmental and localized tandem duplication events. Promoter analysis identified stress- and phytohormone-responsive *cis*-acting elements, including ABRE, MBS, and MeJA-associated motifs. Public transcriptome datasets revealed diverse OsDUF1645 expression patterns under abiotic and hormonal treatments. Integration with the Quantitative Trait Loci Annotation Rice Online (Q-TARO) QTLome identified physical co-localization of multiple OsDUF1645 loci with stress- and agronomic-trait QTLs, including salinity-, drought-, root architecture-, and water-deficit-associated regions. On Chromosome 1, *OsDUF1645.1*, *OsDUF1645.2*, *OsDUF1645.3*, and *OsDUF1645.4* overlapped QTL intervals associated with salinity-related physiological traits, including Na^+^ uptake and Na^+^ balance, and drought-related root traits. On Chromosome 5, the tandemly arranged *OsDUF1645.8*, *OsDUF1645.9*, and *OsDUF1645.10* co-localized with QTLs related to root architecture and water-deficit responses. qRT-PCR validation under salinity, osmotic stress, and cadmium exposure confirmed distinct stress-responsive expression profiles; *OsDUF1645.6* exhibited broad multi-stress responsiveness, whereas *OsDUF1645.3* was downregulated under several conditions. **Conclusions**: The OsDUF1645 family exhibits substantial functional diversification, supported by distinct regulatory architectures, expression profiles, and QTL associations. These findings provide a framework for prioritizing OsDUF1645 candidates for functional validation and their potential application in molecular breeding and development of climate-resilient rice cultivars.

## 1. Introduction

Research on gene families, including their structure, function, and evolution, provides important insights into plant development and adaptation to environmental changes [1]. With the rapid development of genomics and proteomics, numerous previously unknown gene families have been identified. Among these, Domains of Unknown Function (DUFs) are conserved protein families whose biological roles are poorly characterized [2]. According to Pfam release 35.0, 4795 DUF families have been identified, accounting for approximately 24% of the 19,632 entries in the database, and most of them remain poorly characterized [3].

DUF families are highly conserved across diverse organisms and are widely distributed in animals, plants, and fungi. This evolutionary conservation implies the involvement of members of these families in various pivotal cellular processes; investigating such genes can help deduce the biochemical functions of unidentified proteins within highly divergent lineages, based on the conserved domains defining these family branches. Increasing efforts have been made to investigate the biological functions of DUF-containing genes in different species [4,5]. Existing studies have shown that DUF proteins play important biological roles in plant growth and development, as well as in responses to biotic and abiotic stresses [6,7,8,9]. For instance, the DUF647 protein Root UVB Sensitive 1 (RUS1) regulates ultraviolet-B sensing during early seedling morphogenesis [10]. The DUF283 domain of Dicer proteins has been reported to possess a double-stranded RNA-binding fold, suggesting a potential functional role in target selection during small RNA processing [11,12]. While core Dicer-like proteins cleave RNA precursors, accessory RNA-binding proteins interact with these sequences to fine-tune small RNA abundance and post-transcriptional silencing efficacy [13]. In rice, these processed microRNAs and small interfering RNAs form active RNA-induced silencing complexes (RISCs) with ARGONAUTE proteins, repressing target genes to coordinate hormone signaling, growth, and innate immunity [14]. Crucially, these pathways trigger epigenetic modifications via RNA-directed DNA methylation (RdDM), suppressing transposable elements and modulating gene expression under environmental stress [15].

Therefore, characterizing conserved, uncharacterized lineages like the rice OsDUF1645 family within these regulatory frameworks is a vital prerequisite to uncovering novel multi-omics mechanisms governing crop adaptation. In addition, some members of the DUF579 family play multiple roles in cell wall biosynthesis [16,17]. Specifically, DUF579 proteins act as essential components of multi-protein Golgi complexes that interact directly with glycosyltransferases to synthesize the xylan backbone, while other members are responsible for the 4-O-methylation of glucuronic acid on arabinogalactan proteins [18,19]. These specialized hemicellulosic and glycoprotein modifications dynamically strengthen the secondary cell wall matrix, fortifying mechanical barriers to protect plant tissues against environmental osmotic changes, water-deficit pressures, and pathogen attacks. Notably, an increasing number of DUF-containing proteins have been implicated in plant stress responses. For example, overexpression of the DUF642 domain-containing protein AhDGR2 increased sensitivity to NaCl treatment in transgenic *Arabidopsis* [7]. In rice, *OsSIDP366*, a DUF1644-containing gene, has been reported to function as a regulator of processing bodies (PBs) and stress granules (SGs) and positively regulate responses to drought and salt stresses [20]. Likewise, DUF4228 family members in *Arabidopsis* respond to osmotic, cold, and salt treatments [21], whereas *OsDSR2*, encoding a DUF966-containing protein in rice, negatively regulates responses to salt stress, PEG-induced osmotic stress, and ABA signaling, providing useful evidence for understanding the functional roles of DUF966 genes in plant responses to abiotic stress [22]. Furthermore, diverse uncharacterized lineages have been functionally categorized based on their specific stress-adaptive roles, separating into structural metabolic components and localized signaling nodes. Specifically, proteins embedding DUF21 and DUF569 domains operate as systemic transport and developmental regulators; for instance, DUF21 members form multi-domain complexes alongside cystathionine-β-synthase blocks to govern ion flux, while specialized DUF569 genes act as positive upstream elements triggering drought-responsive morphological changes [23,24]. Conversely, members containing DUF936 and DUF315 domains function as key metabolic and redox homeostatic regulators, with targets like OsSSID6 negatively modulating salinity tolerance in rice by coordinating metabolic flux and reactive oxygen species (ROS) scavenging arrays [25,26].

Although several DUF families have now been functionally characterized, the biological functions and evolutionary diversification of DUF1645-containing proteins remain comparatively poorly understood. Increasing evidence, however, suggests that DUF1645 proteins may participate in both abiotic stress adaptation and agronomically important developmental processes. In rice, *OsSGL*, which encodes a DUF1645-containing protein, was reported to enhance drought tolerance when overexpressed in rice and *Arabidopsis*, indicating a potential role for DUF1645 in drought-responsive pathways [27]. Subsequent comparative studies in Poaceae further demonstrated that orthologs of *OsSGL* can positively influence drought tolerance as well as grain length and grain weight in rice, suggesting that DUF1645 proteins may have functions extending beyond stress adaptation to include yield-related developmental processes [28]. Notably, these phenotypic improvements exhibit strong environmental consistency, maintaining stable improvements in both survival metrics and yield architecture across controlled growth chambers, greenhouse settings, and multi-locational field trials under fluctuating water-deficit pressures. In maize, ectopic expression of *ZmDUF1645* was reported to increase grain length and yield, whereas its effect on drought tolerance differed from that observed for *OsSGL* in rice [29]. These contrasting phenotypic effects among homologous DUF1645 genes suggest that, despite their evolutionary conservation, individual family members may have undergone functional diversification following gene duplication and speciation. However, the evolutionary relationships of the DUF1645 family have not yet been systematically explored, and the characteristics and functions of most members remain unclear. In particular, it remains unclear how the complete set of OsDUF1645 genes is organized in the current rice reference genome, how their evolutionary relationships and duplication history relate to potential functional divergence, and whether individual members are preferentially associated with particular abiotic stress responses or agronomic traits. Furthermore, the physical relationships between OsDUF1645 loci and previously mapped rice QTLs have not been systematically evaluated. This knowledge gap directly limits downstream translational applications, as the lack of a structured, multi-omics classification framework prevents breeders from precisely prioritizing candidate genes or predicting functional redundancy, thereby hindering the target selection necessary for marker-assisted breeding and precision genome editing in rice. Integrating genome-wide family characterization with evolutionary analysis, stress-responsive expression profiling, and QTL interval co-localization may therefore provide a more comprehensive framework for prioritizing candidate OsDUF1645 genes and understanding their potential functional diversification. Rice is continuously exposed to a wide range of environmental constraints, such as osmotic stress, heavy-metal toxicity, salinity, temperature fluctuations, and drought. The identification of genes associated with stress-adaptive and agronomic traits are therefore essential for developing climate-resilient rice cultivars. Quantitative Trait Loci (QTLs) provide an important genetic framework for linking genomic regions with complex phenotypes, but most QTL intervals encompass large genomic regions containing numerous candidate genes. Consequently, integrating QTL information with the physical positions of candidate genes can provide an additional layer of evidence for prioritizing genes potentially involved in stress adaptation and agronomic performance. 

Therefore, this study aimed to provide a comprehensive genome-wide characterization of the DUF1645 gene family in the current rice reference genome. We systematically identified and curated OsDUF1645 family members and investigated their physicochemical characteristics, gene structures, chromosomal distribution, phylogenetic relationships, and duplication and collinearity patterns. Promoter *cis*-acting elements were further analyzed to assess their potential regulatory responses to abiotic stresses and phytohormones. To investigate potential functional diversification among family members, we characterized their expression patterns across tissues, developmental stages, abiotic stress conditions, and hormone treatments using publicly available transcriptomic datasets, followed by qRT-PCR validation under selected stress conditions. In addition, we integrated the physical genomic positions of *OsDUF1645* genes with rice QTL intervals from Q-TARO to identify candidate co-localizations with stress-related and agronomic traits. Collectively, this integrated genomic, evolutionary, transcriptomic, experimental, and QTL-based analysis was designed to clarify the organization and potential functional diversification of the OsDUF1645 family and to provide candidate genes and genomic evidence for future functional studies and stress-resilient rice improvement.

## 2. Materials and Methods

### 2.1. Identification of OsDUF1645 Genes in Rice

To identify OsDUF1645 genes in rice, genomic and proteomic sequences of *O. sativa*, *Zea mays*, and *Arabidopsis thaliana* were retrieved from the Ensembl Plants database (http://plants.ensembl.org/, accessed on 20 February 2025). The Hidden Markov Model (HMM) profile corresponding to the DUF1645 domain (Accession: PF07816) was downloaded from the Pfam database (http://pfam.xfam.org/, accessed on 24 February 2025) and used as a query to scan protein sequence datasets with HMMER v3.3 (hmmer.org) using an E-value threshold of <10^−5^. To accurately map unique gene loci and prevent artificial inflation of the family size, alternative splicing variants were resolved by executing a comprehensive cross-reference script against the official Rice Annotation Project (RAP-DB) and Rice Genome Annotation Project (RGAP) database structural models. Splice variants sharing a common primary transcription start site or locus origin were filtered out by retaining exclusively the longest representative protein sequence for each distinct locus, while shorter non-homologous isoforms or completely independent overlapping open reading frames were cross-checked manually via InterProScan to ensure no unique functional sub-domains or multi-domain architectural variants were prematurely excluded. The remaining candidate sequences were subjected to structural validation using the SMART (https://smart.embl.de/smart/change_mode.cgi, accessed on 10 March 2025), Pfam (http://pfam.xfam.org/, accessed on 10 March 2025), and the NCBI Conserved Domain Database (CDD) (https://www.ncbi.nlm.nih.gov/Structure/cdd/cdd.shtml, accessed on 10 March 2025) to confirm the presence of the DUF1645 domain; any sequences with highly truncated, corrupted, or absent domains were strictly discarded. Discrepancies in domain boundaries between databases were resolved by prioritizing consensus predictions or the more stringently defined CDD models. Finally, the primary sequence data of the validated OsDUF1645 proteins (Table S1) were analyzed using the ExPASy ProtParam tool (https://web.expasy.org/protparam/, accessed on 15 March 2025) to calculate fundamental physicochemical properties, including total amino acid length, molecular weight (MW), and theoretical isoelectric point (pI).

### 2.2. Phylogenetic Analysis, Gene Structure and Conserved Motif Analysis

For phylogenetic analysis, full-length protein sequences or their extracted conserved DUF1645 domains from *O. sativa*, *Z. mays*, and *A. thaliana* were subjected to multiple sequence alignment (MSA) using the ClustalW algorithm in MEGA 11.0 (https://www.megasoftware.net/, accessed on 20 July 2025). The selection of ClustalW was methodologically justified by the structural architecture of the target proteins; the identified DUF1645 family members represent entirely intronless, single-domain protein structures lacking highly variable, long non-homologous extensions or unstructured flanking loop regions. Because the target family is characterized by high sequence conservation and minimal gap penalties across divergent plant lineages, the progressive alignment strategy of ClustalW provided an optimal balance of structural alignment precision and computational efficiency without introducing the artificial gap-insertion biases often associated with complex heuristic or iterative alignment algorithms. A phylogenetic tree was subsequently constructed using the Neighbor-Joining (NJ) method. Genetic distances were calculated using the p-distance model, and positions containing gaps or missing data were handled via pairwise deletion. To assess the topological reliability of the branching patterns, a bootstrap analysis with 1000 pseudo-replicates was executed. The final phylogenetic tree was visualized and stylistically edited using the Interactive Tree Of Life (iTOL v6, https://itol.embl.de/, accessed on 25 July 2025). To investigate the structural diversity of the OsDUF1645 genes, exon–intron organizations were determined by comparing full-length coding sequences (CDSs) with their corresponding genomic DNA sequences using the Gene Structure Display Server 2.0 (GSDS; https://gsds.gao-lab.org/, accessed on 25 April 2025). To ensure absolute accuracy in structural model plotting, exon–intron boundary validation was rigorously reinforced by requiring a minimum of 98.0% sequence identity and 100% query coverage across the full length of the matched transcript tracks. Furthermore, the structural junctions of any flanking untranslated regions (UTRs) and internal splice boundaries were independently cross-checked against the definitive Rice Annotation Project (RAP-DB) genome coordinates, systematically verifying the invariant presence of the canonical GT-AG splice donor/acceptor dinucleotide consensus sequence at all mapped intron borders to eliminate any automated machine-splicing prediction artifacts. Conserved de novo motifs within the OsDUF1645 protein sequences were identified using the Multiple Em for Motif Elicitation tool (MEME Suite v5.5.0; https://meme-suite.org/meme/tools/meme, accessed on 28 April 2025). The detection parameters were configured to detect up to 10 distinct motifs, according to the approach adopted in a previous study [30]. The final integration of the phylogenetic tree, gene structures, and conserved motif architectures was rendered for publication using TBtools-II (version 2.121) software [31].

### 2.3. Chromosomal Localization and Gene Duplication Analysis

To determine the physical distribution of the OsDUF1645 genes, exact chromosomal coordinates and locus identifiers were extracted from the rice genome General Feature Format (GFF3/GTF) annotation file downloaded from Ensembl Plants (http://plants.ensembl.org/index.html, accessed on 13 March 2025). To guarantee absolute spatial positioning accuracy and resolve database fragmentation, all extracted coordinate ranges, strand orientations, and structural locus boundaries were systematically cross-checked and harmonized across both the Rice Annotation Project Database (RAP-DB; IRGSP-1.0) and the Rice Genome Annotation Project (RGAP; MSU RG7). Structural exceptions between the two nomenclature streams were resolved manually: loci missing from RAP-DB but maintained in RGAP (specifically OsDUF1645.4/LOC_Os01g47270) and loci exclusive to RAP-DB but absent from RGAP (specifically *OsDUF1645.14*/*Os12g0556900*) were successfully preserved based on their verified physical domain coordinates. The finalized, cross-validated physical positions of the candidate genes were visually mapped across rice chromosomes using TBtools-II software, with actual nucleotide scales. To systematically trace evolutionary origins and duplication mechanisms, an all-against-all local sequence alignment of the entire rice proteome was performed using BLASTP (BLAST+ V2.17.0) with an E-value cutoff of E < 10^−5^. The resulting BLASTP alignment matches and the genomic coordinate files were integrated into the Multiple Collinearity Scan toolkit (MCScanX) [32] to identify syntenic OsDUF1645 gene pairs and explicitly classify gene duplication modalities into tandem, segmental/whole-genome, proximal, or dispersed events based on default spatial block distance and gene density thresholds. To ensure reproducibility and remove false positives, the identified paralogous relationships and syntenic blocks were independently cross-validated using a dual-verification loop by verifying that the flanking anchoring genes within a 10-gene upstream/downstream window maintained significant collinearity metrics, and subsequently executing a reciprocal best-hit BLASTP check between the core duplicated paralogs to confirm shared evolutionary homology. Finally, the physical positions of the OsDUF1645 genes, along with the syntenic connecting lines that track segmentally duplicated paralogous pairs across the genome, were rendered in a multi-layered layout using Circos v0.69 software [33]. 

### 2.4. In Silico Mapping and Co-Localization Within the Rice Stress QTLome

The genomic distribution of the OsDUF1645 gene family was evaluated by physical co-localization with previously reported rice Quantitative Trait Loci (QTLs) using the Q-TARO database available through RAP-DB. Genomic coordinates of OsDUF1645 loci were obtained from the IRGSP-1.0/MSU RG7 reference annotation and standardized to chromosome, start, end, and midpoint positions. QTL records were retrieved with their identifiers, genomic intervals, associated traits, and functional annotations. QTL intervals with inconsistent coordinate ordering were corrected by assigning the smaller coordinate as the start position and the larger coordinate as the end position. Physical co-localization was defined when the genomic midpoint of an OsDUF1645 locus was contained within a reported QTL interval (QTLstart ≤ Geneposition ≤ QTLend). No additional flanking distance or proximity threshold was applied. All QTLs satisfying this criterion were retained for each gene, and the resulting annotations included QTL number, identifiers, names, reported traits, chromosome, and functional class. Functional classes were subsequently grouped into broader categories, including drought/soil stress, salinity, cold stress, and other agronomic or stress-related traits. Genomic relationships were visualized using a chromosome-scale ideogram displaying all 12 rice chromosomes, OsDUF1645 positions, and their corresponding QTL intervals. A Sankey diagram was additionally generated to summarize the relationships among chromosome, OsDUF1645 gene, QTL functional class, and individual QTL features.

### 2.5. Analysis of Cis-Acting Elements in Promoter Regions

Promoter sequences corresponding to the OsDUF1645 family were obtained from the RAP-DB IRGSP-1.0 rice genome annotation as 2 kb upstream sequences relative to the start codon ATG. These RAP-DB promoter sequences were processed to retain the proximal 1500 bp upstream region (−1500 bp to −1 bp relative to ATG) for downstream cis-regulatory analysis. A genome-wide background set of 1000 rice promoters was randomly sampled from the same RAP-DB promoter collection after excluding all OsDUF1645 family promoters. Background sequences were processed identically by extracting the corresponding 1500 bp upstream regions to ensure comparable promoter lengths and coordinate systems between OsDUF1645 and background datasets. Both OsDUF1645 and background promoter sequences were analyzed using PlantCARE (V2.0, https://bioinformatics.psb.ugent.be/webtools/plantcare/html/, accessed on 10 November 2025) to identify annotated *cis*-acting regulatory elements (CAREs). PlantCARE outputs from background promoter batches were merged, duplicate annotations were removed, promoter identifiers and genomic coordinates were standardized, and invalid annotations were discarded. The excluded *OsDUF1645.4* entry, which lacked a valid RAP-DB promoter assignment, was removed before downstream analyses. For CARE enrichment analysis, PlantCARE annotations were converted into promoter-level binary presence/absence matrices, where rows represented individual promoters and columns represented individual CARE types. Only CAREs detected in both OsDUF1645 and background promoter datasets were retained for statistical testing. For each CARE, a 2 × 2 contingency table was constructed comparing CARE presence and absence between OsDUF1645 promoters and background promoters. Fisher’s exact test with a one-sided alternative hypothesis was applied to identify CAREs preferentially enriched in OsDUF1645 promoters. Multiple testing correction was performed using the Benjamini–Hochberg method. CAREs were considered significantly enriched when they exhibited an FDR < 0.05 and an enrichment ratio > 1. Enrichment strength was quantified using prevalence ratios, odds ratios, and log2-transformed enrichment values. CARE abundance architecture was further evaluated by calculating the copy number of each CARE within individual promoters. Mean and total CARE copy numbers were compared between OsDUF1645 and background groups, and relative copy-number enrichment was calculated as the ratio between group-specific mean CARE counts. To examine positional organization, CARE annotations were classified into three promoter intervals according to their ATG-relative coordinates: distal (−1500 to −1001 bp), intermediate (−1000 to −501 bp), and proximal (−500 to −1 bp). CARE density and distribution patterns across these regions were summarized to characterize promoter regulatory topology. A TBtools-style promoter architecture map was generated to visualize the spatial organization of OsDUF1645 CAREs. Each promoter was represented as a 1.5 kb horizontal track, with individual CAREs positioned according to their upstream coordinates. CAREs were displayed according to element identity and annotated with functional categories. To ensure reproducibility of functional classification, CAREs were assigned to regulatory groups using a predefined annotation-based framework derived from PlantCARE descriptions. Specifically, CAREs containing terms associated with abscisic acid (ABA), auxin, gibberellin, methyl jasmonate (MeJA), salicylic acid, or other hormone-responsive elements were classified as hormone-responsive CAREs. CAREs annotated with stress-associated terms, including drought, low temperature, anaerobic induction, defense, pathogen response, or abiotic stress responsiveness, were assigned to the stress-responsive CARE category. CAREs containing MYB-, MYC-, bZIP-, or other transcription factor binding annotations were classified as transcription-factor regulatory elements. Elements associated with chloroplast/light regulation, developmental regulation, meristem activity, seed-specific expression, or growth-related functions were grouped as development/light-associated CAREs. CAREs without a clear functional description in PlantCARE were retained as unknown/other regulatory elements and were not assigned to specific biological categories. This rule-based classification was applied consistently across OsDUF1645 and background promoter datasets. Finally, CARE identity, abundance, functional category, and positional distribution were integrated into a CARE copy-number heatmap and promoter architecture visualization to provide a comprehensive representation of OsDUF1645 *cis*-regulatory organization.

### 2.6. Expression Profiling and Integrated Stress-Response Analysis of OsDUF1645 Genes

To characterize transcriptional responses of the OsDUF1645 family under environmental and hormonal perturbations, publicly available rice transcriptome datasets were mined from the Transcriptome Encyclopedia of Rice database (TENOR; https://rapdb.dna.naro.go.jp/TENOR_in_RAP-DB.html, accessed on 26 March 2025) using corresponding locus identifiers from the Rice Annotation Project Database (RAP-DB; https://rapdb.dna.naro.go.jp, accessed on 26 March 2025). The data used correspond to the DDBJ Sequence Read Archive (DRA)-hosted DRA000159 for high-salinity- and DRA001092 for high-cadmium-induced stresses, and DRA000959 for other stresses. Expression profiles were retrieved across a comprehensive panel of abiotic stress and phytohormone treatments, including salinity, cadmium, drought, cold, osmotic stress, abscisic acid (ABA), and jasmonic acid (JA) responses, covering multiple tissues (shoot and root) and time points ranging from early responses (1–12 h) to prolonged stress responses (1–10 d). TENOR expression profiles represent curated reference expression tracks generated from experimentally validated rice transcriptome datasets with biological cohorts integrated at each treatment/time-point combination. Because the present study used the TENOR consensus expression framework rather than reprocessing individual RNA-seq libraries, replicate-level expression values were not independently recalculated. Instead, the available TENOR consensus profiles derived from biological experimental designs were used for comparative stress-response mining. The corresponding treatment metadata and experimental information were retained during dataset integration. Expression values were organized into a unified OsDUF1645 stress-response matrix containing treatment, tissue, and temporal information. Relative transcriptional responses were evaluated using log2 fold-change (Log2FC) values compared with corresponding untreated controls. For classification purposes, transcriptional activation was defined as Log2FC ≥ 1.0, representing at least a two-fold induction, whereas repression was defined as Log2FC ≤ −1.0. Genes showing activation across three or more independent stress or hormone categories were classified as broad-spectrum or multi-stress-responsive candidates, whereas genes activated under only one or a limited number of treatment categories were considered condition-specific responders. These predefined thresholds provided reproducible criteria for distinguishing general stress-associated transcriptional responses from specialized environmental responses. Temporal response patterns were further evaluated by separating early inducible responses (1–12 h) from late responses (1–10 d). Early induction profiles were used to identify genes potentially involved in rapid stress perception or signaling, whereas persistent activation across extended exposure periods was interpreted as a potential contribution to long-term adaptation processes. In parallel, repression events were quantified by calculating the number of downregulated treatment responses, affected stress categories, mean negative Log2FC values, and minimum observed Log2FC values. These repression-associated parameters were incorporated as a penalty factor during integrated candidate prioritization to distinguish genes dominated by activation responses from those exhibiting widespread transcriptional suppression. Potential hormone–stress regulatory interactions were examined by comparing ABA- and JA-responsive expression profiles with abiotic stress datasets. Binary response matrices were constructed to identify genes showing overlapping activation patterns between hormone treatments and environmental stresses, allowing detection of candidate ABA-associated and JA-associated regulatory modules. Finally, TENOR-derived expression features were integrated with promoter *cis*-regulatory information and QTL annotations. Expression-derived parameters, including stress breadth, activation strength, temporal response behavior, and repression tendency, were combined with PlantCARE *cis*-element enrichment profiles and genomic QTL associations to establish an integrated candidate ranking framework for OsDUF1645 genes. This combined approach enabled prioritization of family members supported simultaneously by transcriptional responsiveness, promoter regulatory potential, and genetic evidence associated with stress adaptation. All data extraction, matrix integration, response classification, statistical evaluation, and candidate scoring procedures were performed in R (version 4.6.1) using tidyverse-based workflows.

### 2.7. Plant Materials, Growth Conditions, and Stress Treatments

*O. sativa* L. ssp. *japonica* cv. ‘Nipponbare’ was used as the experimental material. Clean seeds were surface-sterilized with a 2.5% (*v*/*v*) sodium hypochlorite (NaClO) solution for 30 min, thoroughly rinsed with deionized water, and soaked in the dark at 30 °C for 48 h to ensure uniform germination. Uniformly germinated seeds were transferred to a hydroponic system, cultured in 25% strength Hoagland nutrient solution for 3 days, and then acclimated in 50% strength Hoagland solution. Hydroponic seedlings were cultivated in a controlled-environment growth chamber at 28 °C (day)/25 °C (night) with a 14 h light/10 h dark photoperiod and 70% relative humidity.

Upon reaching the two-week-old seedling stage, uniform plants were subjected to abiotic stress treatments. Salt stress was imposed by transferring seedlings into a fresh Hoagland solution supplemented with 200 mM NaCl. Although this NaCl concentration represents a relatively severe salinity challenge compared with typical field conditions, it has been widely applied in controlled rice seedling experiments to generate reproducible osmotic and ionic stress responses and to activate stress-responsive transcriptional programs within a short experimental timeframe. Osmotic stress was induced using Hoagland solution containing 20% (*w*/*v*) Polyethylene Glycol 6000 (PEG 6000; Sangon biotech, Shanghai, China), which was used to simulate water-deficit conditions by reducing external water potential without introducing ionic toxicity. Leaf tissues from treated and corresponding mock-treated control plants were harvested at specific intervals (0, 3, 6, 9, 12, and 24 h post-treatment). The selected sampling scheme was designed to capture early transcriptional signaling events occurring within the first hours after stress exposure (3–12 h), including rapid activation of stress-responsive pathways, while the 24 h time point represented an early adaptive response phase following sustained stress exposure. For each biological replicate, leaf blades from three independent seedlings were pooled together to minimize inter-individual variation. All collected tissue samples were flash-frozen in liquid nitrogen immediately upon harvest and stored at −80 °C for subsequent downstream molecular analysis. Every experimental group comprised three completely independent biological replicates generated from separately handled plant materials. These biological replicates represented independent experimental units rather than technical repetitions, ensuring reproducibility of the observed transcriptional responses.

### 2.8. Total RNA Extraction, cDNA Synthesis, and qRT-PCR Analysis

Total RNA was isolated from frozen rice leaf tissues using the KK Fast Plant Total RNA Kit (Cat. No. ZP405K; Zomanbio, Beijing, China) according to the manufacturer’s instructions. RNA concentration and purity were assessed using a NanoDrop 2000 spectrophotometer (Thermo Fisher Scientific, Wilmington, DE, USA), and only samples with stable A260/A280 ratios between 1.8 and 2.1 were used for downstream analysis. Residual genomic DNA contamination was removed, and first-strand cDNA synthesis was performed from 1 μg of total RNA using the EX RT Kit First-Strand cDNA Synthesis Kit with gDNA Remover (Cat. No. ZR108; Zomanbio, Beijing, China). The reverse transcription reaction incorporated a combination of Oligo (dT) and random hexamer primers to provide broad transcript coverage and improve reverse transcription consistency. Quantitative real-time PCR (qRT-PCR) was performed in a total reaction volume of 10 µL containing 5 µL of 2× HQ SYBR qPCR Mix (High ROX) (Cat. No. ZF503; Zomanbio, Beijing, China), 0.2 µL each of gene-specific forward and reverse primers (10 µM), 1.0 µL of five-fold diluted cDNA template, and 3.6 µL of nuclease-free water. Amplification was performed using an ABI StepOne Real-Time PCR System (Applied Biosystems, Foster City, CA, USA). The thermal cycling program consisted of an initial denaturation step at 95 °C for 30 s, followed by 40 cycles of 95 °C for 15 s and 60 °C for 30 s. The annealing temperature was optimized individually for each primer pair using gradient PCR analysis across a temperature range of 55–65 °C, and 60 °C was selected as the optimal annealing temperature based on maximal amplification specificity and minimal nonspecific products. Following amplification, melting curve analysis was performed from 60 °C to 95 °C to verify amplification specificity and exclude primer-dimer formation. To ensure accurate normalization, candidate reference genes were initially evaluated across all stress treatments and sampling time points. Relative transcript abundance was calculated using the comparative 2^−ΔΔCt^ method [34]. For each biological replicate, Ct values obtained from three technical replicates were averaged before calculation of ΔCt and ΔΔCt values. All expression values represent the mean of three independent biological replicates, with each biological replicate analyzed using three technical replicates. The list of primers used are listed in Table S2.

### 2.9. Statistical Analysis

All qRT-PCR data were analyzed using R statistical software. Data are presented as mean ± standard error of the mean (SEM) from three independent biological replicates (*n* = 3). Before statistical testing, datasets were evaluated for distribution characteristics and homogeneity of variance to assess their suitability for parametric analysis. For each OsDUF1645 gene within each stress condition, differences in relative expression among the 0, 3, 6, 9, 12, and 24 h time points were assessed using one-way analysis of variance (ANOVA), followed by Tukey’s honestly significant difference (HSD) multiple comparison test. Statistical significance was defined as *p* < 0.05. For graphical presentation, bars represent mean expression levels with error bars indicating SEM. Tukey’s compact-letter display was used to indicate statistical differences among time points: groups sharing the same letter were considered not significantly different, whereas groups assigned different letters were considered significantly different (*p* < 0.05). Figures were generated in R using the ggplot2 package. The R scripts and datasets supporting the analyses are available from the corresponding author upon reasonable request.

## 3. Results

### 3.1. Identification and Characterization of DUF1645 Genes in Rice

A genome-wide analysis for the identification of DUF1645 gene family members in rice using a Hidden Markov Model (HMM) search targeting the characteristic DUF1645 domain (Accession: PF07816), followed by rigorous structural validation against the NCBI Conserved Domain Database (CDD), SMART, and Pfam databases, allowed the identification of a total of 14 non-redundant OsDUF1645 genes. Sequences lacking an intact or verifiable DUF1645 domain were systematically excluded. These 14 verified genes were sequentially designated as *OsDUF1645.1* through *OsDUF1645.14* based on their respective physical distributions and locus configurations within the rice genome (Table 1). The relatively limited number of OsDUF1645 members compared with other larger DUF families likely reflects the evolutionary conservation and restricted expansion of this specific DUF lineage in rice, as only proteins containing the conserved DUF1645 domain and meeting the established identification criteria were retained.

Primary sequence and physicochemical analysis revealed substantial structural diversity among the identified OsDUF1645 proteins. The polypeptide lengths ranged from 231 to 430 amino acids (aa), corresponding to predicted molecular weights (MW) spanning from 24.05 kDa to 44.59 kDa. The theoretical isoelectric points (pI) exhibited a wide distribution, ranging from 5.48 to 11.65. Specifically, three members (OsDUF1645.4, OsDUF1645.8, and OsDUF1645.10) were characterized as acidic proteins (pI < 7.0), whereas the remaining 11 members displayed strongly basic properties (pI > 7.0). This pronounced variation in pI values suggests potential functional diversification among OsDUF1645 proteins, as differences in surface charge may influence protein stability, molecular interactions, and association with negatively charged cellular components. Moreover, the distinct charge properties of OsDUF1645 members may contribute to differences in subcellular targeting or compartment-specific interactions, since proteins enriched in basic residues often exhibit enhanced interactions with nucleic acids or acidic cellular structures, whereas more acidic proteins may favor alternative molecular environments. Among the family members, OsDUF1645.6 was the largest protein, with a molecular weight of 44.59 kDa, while OsDUF1645.8 was the smallest, at 24.05 kDa (Table 1). The observed variation in protein size among OsDUF1645 members may reflect evolutionary diversification within this family, potentially arising from sequence insertion/deletion events, domain extension, or lineage-specific modifications during rice evolution. Such structural variation may contribute to functional specialization by affecting protein stability, interaction capacity, or regulatory properties among different family members.

### 3.2. Phylogenetic Relationship Analysis of DUF1645 Proteins from Rice, Arabidopsis and Z. mays

**Figure 1:**
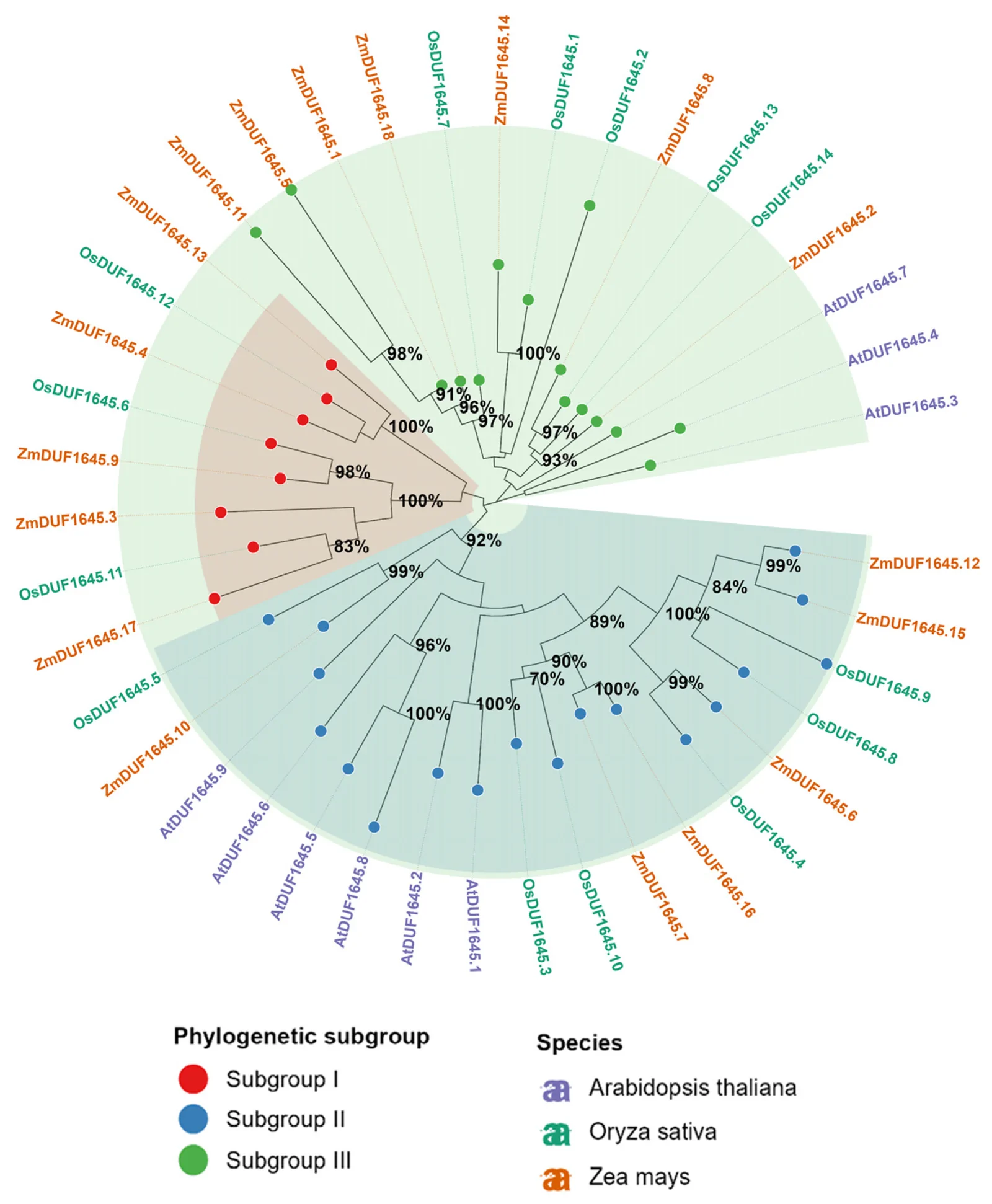
Phylogenetic analysis of DUF1645 proteins from *O. sativa*, *A. thaliana*, and *Z. mays*. The phylogenetic tree was constructed based on MUSCLE alignment of conserved DUF1645 domain sequences using the Neighbor-Joining (NJ) method implemented in MEGA version 11.0 with 1000 bootstrap replicates. Bootstrap values above 75% are indicated at branch nodes, and branch lengths are proportional to the estimated evolutionary distance based on amino acid substitutions.

To elucidate the evolutionary history and diversification patterns of the DUF1645 family, an unrooted phylogenetic tree was reconstructed using the conserved core domain sequences isolated from *O. sativa* (Os), *Z. mays* (Zm), and *A. thaliana* (At) (Figure 1). The conserved DUF1645 core domain was selected for phylogenetic reconstruction because it represents the evolutionarily constrained region shared among family members, whereas variable terminal regions may introduce alignment uncertainty caused by lineage-specific insertions, deletions, or sequence divergence. Therefore, domain-based analysis provides a robust framework for resolving evolutionary relationships among DUF1645 proteins from distantly related plant species. Topology assessment revealed that the DUF1645 proteins partitioned into three well-supported evolutionary lineages (Subgroups I, II, and III). Subgroup I emerged as the largest clade within the family, comprising 23 members. This lineage comprised seven members from *O. sativa*, twelve from *Z. mays*, and four from *A. thaliana*.

Across the tree topology, the OsDUF1645 proteins consistently clustered in immediate sister-clade relationships with ZmDUF1645 orthologs rather than with their AtDUF1645 counterparts, indicating a significantly higher degree of sequence conservation and shared ancestry between the two monocot crops. For instance, highly resolved orthologous pairs were observed between *OsDUF1645.5* and *ZmDUF1645.10*, *OsDUF1645.6* and *ZmDUF1645.9*, *OsDUF1645.12* and *ZmDUF1645.4*, as well as *OsDUF1645.11* and *ZmDUF1645.17*. Taken together, these results demonstrate that the DUF1645 family has remained relatively conserved since the divergence of monocotyledonous and dicotyledonous lineages, consistent with established angiosperm taxonomy. 

### 3.3. Gene Structure and Conserved Motif Analysis

**Figure 2:**
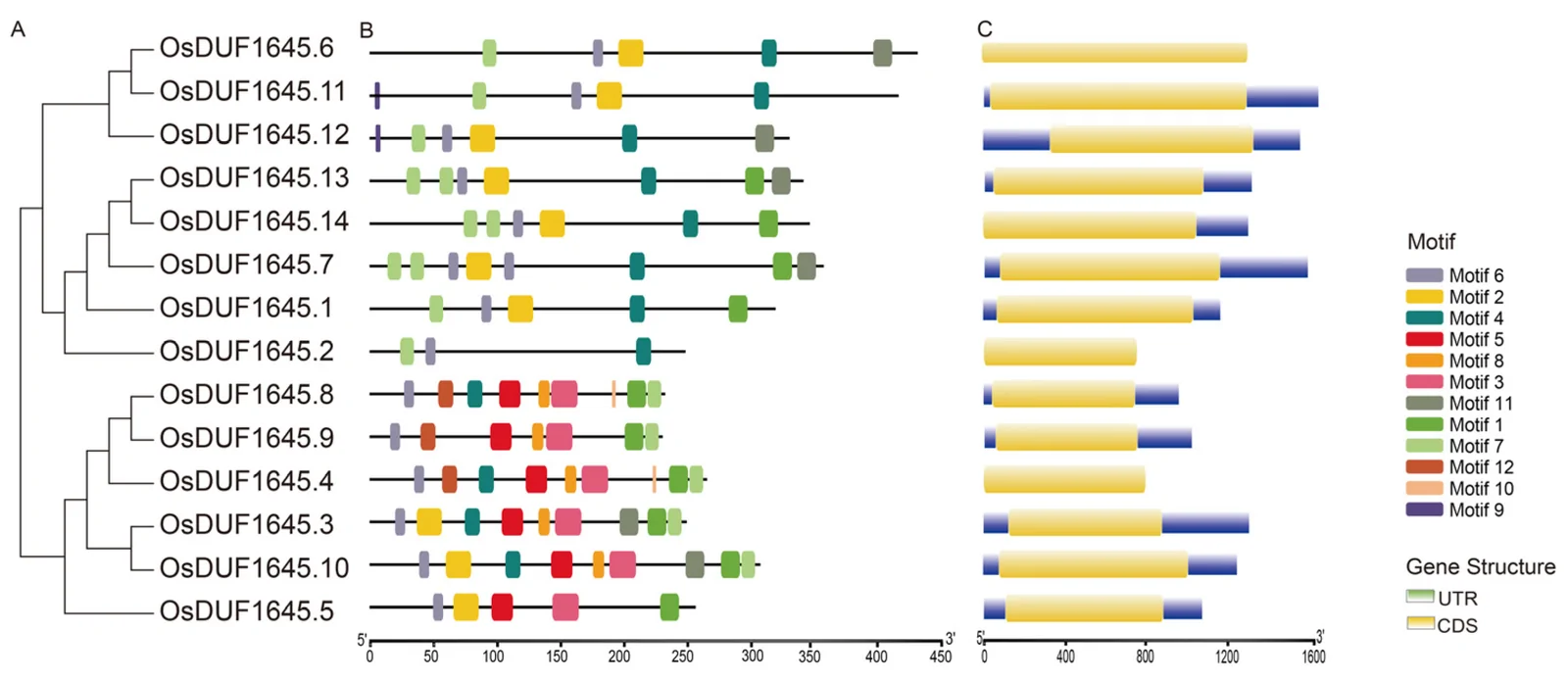
Phylogenetic relationships, conserved motif, and gene structures among the OsDUF1645 proteins. (**A**) The Neighbor-Joining phylogenetic tree was constructed using the full-length OsDUF1645 proteins with MEGA 11.0 software. (**B**) The motif compositions of OsDUF1645 proteins. Ten conserved motifs were represented in the OsDUF1645 proteins; different motifs are displayed in boxes of different colors and numbered. (**C**) Exon–intron structures of the OsDUF1645 genes. Light green boxes represent untranslated 5′ and 3′ regions, yellow boxes represent CDS.

To further evaluate structural diversity and evolutionary relationships among rice DUF1645 proteins, a complementary phylogenetic analysis was performed using full-length amino acid sequences. While the conserved-domain-based phylogeny provides evolutionary resolution based on the shared DUF1645 core region among different plant species, the full-length protein phylogeny incorporates additional variable regions that may contribute to lineage-specific diversification within the rice family. The topology of the full-length OsDUF1645 phylogenetic tree was highly consistent with the multi-species domain-based phylogeny, supporting the reliability of the subgroup classification. The topology of this intra-species tree perfectly mirrored the multi-species clustering pattern, partitioning the 14 OsDUF1645 members into three distinct groups (Groups I, II, and III). The formation of distinct phylogenetic groups, together with group-specific motif arrangements described below, suggests that OsDUF1645 members may have undergone functional diversification during evolution. Divergence among these groups may influence protein interaction networks, regulatory properties, or responses to specific developmental and environmental cues. To uncover hidden structural features within the family, de novo conserved motif discovery was performed using the MEME suite, which identified 10 distinct motifs (designated Motifs 1–10). The spatial distribution and sequential arrangement of these motifs were highly conserved within each specific phylogenetic group (Figure 2), indicating that subgroup formation is supported not only by sequence similarity but also by conserved protein architecture. Conversely, group-specific motif combinations may represent evolutionary modifications that contribute to functional specialization among OsDUF1645 members. The conservation of motif composition and arrangement within phylogenetic groups suggests that these members may retain similar structural frameworks and potentially shared functional properties. Interestingly, members belonging to the same phylogenetic groups displayed comparable patterns of stress-responsive expression in subsequent transcriptomic analyses, suggesting that conserved protein architectures may be associated with coordinated regulatory responses. For instance, Group I members uniformly shared a conserved module consisting of Motifs 6, 2, 4, and 7. Conversely, a complex arrangement of Motifs 1, 2, 4, 6, and 7, was present across Group II members, with the sole exception of *OsDUF1645.2*. Notable variations in motif architecture were also observed; *OsDUF1645.2* exhibited a highly simplified structure, containing a minimal footprint comprising only Motifs 4, 6 and 7. In parallel with the protein architecture analysis, the exon–intron structural configurations of the OsDUF1645 genes were mapped by aligning their coding sequences (CDSs) to the corresponding genomic DNA scaffolds. Remarkably, structural modeling revealed that all 14 OsDUF1645 genes are completely intronless, devoid of any intervening splicing sequences. This uniform single-exon gene organization suggests that the OsDUF1645 family has maintained a high level of genomic structural conservation during rice evolution.

### 3.4. Chromosome Localization, Gene Duplication and Sequence Similarity Analysis

**Figure 3:**
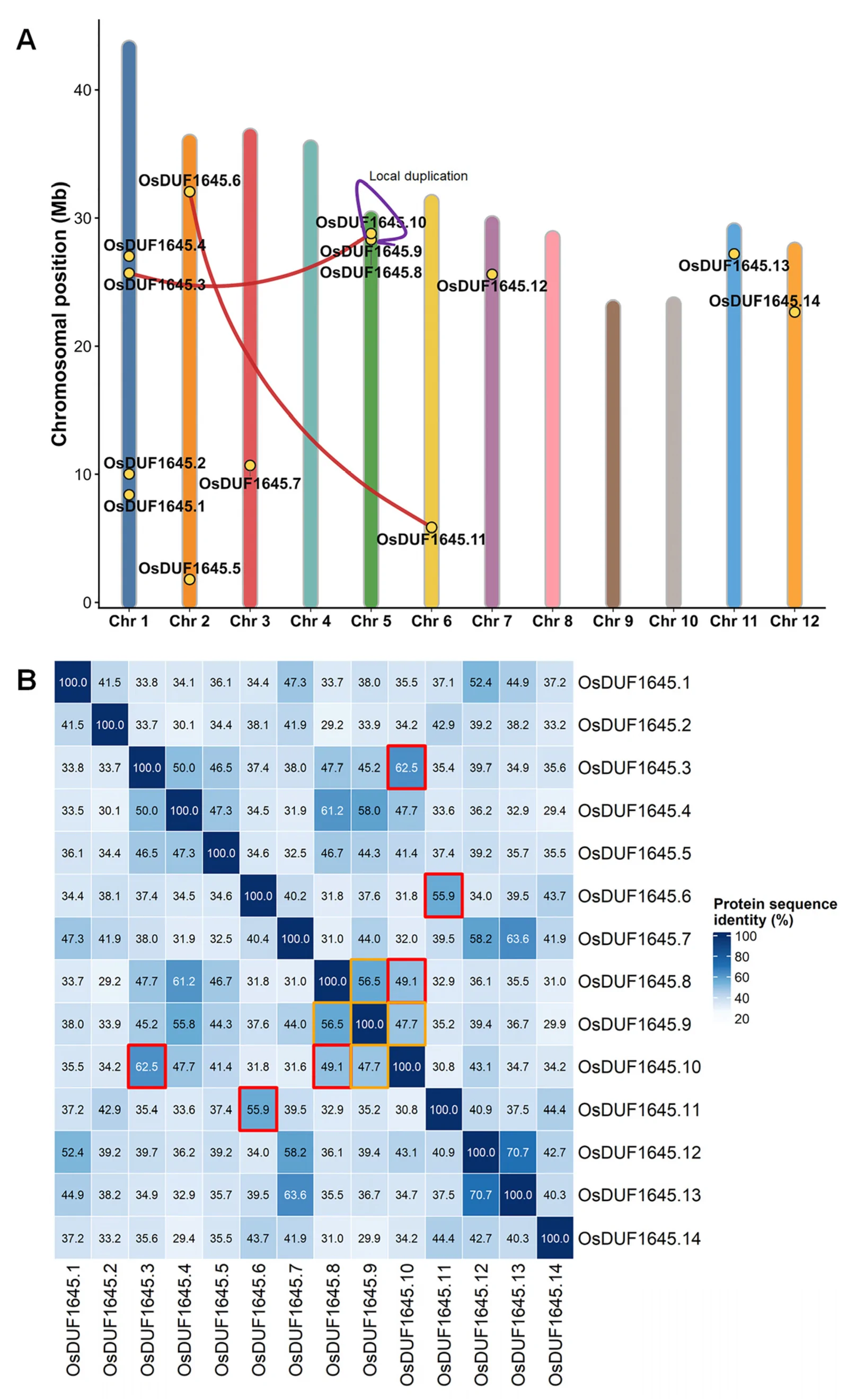
Chromosomal location, gene duplications, and synteny analysis. (**A**) Chromosome localization and collinear relationships of DUF1645 genes in rice. The vertical structural bars represent individual chromosomes mapped according to the uncropped physical coordinates of IRGSP-1.0 reference genome assembly, with length scales denoted in base pairs (Mb). Unique stable locus tags (yellow dots) are anchored to their exact physical mapping coordinates on each track backbone and corresponding gene alias tags are displayed. The red lines indicate inter-chromosomic collinear relationships based the MCScanX synteny analysis results. The purple arc indicates local gene duplication event. Chromosomes without identified family members are retained as essential structural backdrop anchors to maintain global genome transparency. (**B**) Heatmap showing global pairwise sequence similarity of DUF1645 genes in rice. Pairwise global amino-acid sequence identity (%) was calculated using BLOSUM62-based global alignment. Synteny-supported paralogous pairs are outlined in red, while the tandemly duplicated cluster is annotated separately.

To determine physical distribution patterns, the genomic coordinates of the 14 OsDUF1645 genes were mapped across the native rice chromosomes. The chromosomal positions and genomic coordinates of OsDUF1645 members were cross-validated against the IRGSP-1.0 rice genome annotation to ensure consistency and accuracy of gene localization based on RAPDB and RGAP. The family members exhibited an asymmetrical distribution across eight of the 12 rice chromosomes (Figure 3A). Chromosome 1 contained the highest density with four genes (*OsDUF1645.1*, *OsDUF1645.2*, *OsDUF1645.3*, and *OsDUF1645.4*), followed closely by Chromosome 5 with three genes (*OsDUF1645.8*, *OsDUF1645.9*, and *OsDUF1645.10*). The localized accumulation of OsDUF1645 genes on Chromosomes 1 and 5 suggests that these genomic regions may represent evolutionary hotspots for family member retention or expansion. Chromosome 2 harbored two family members (*OsDUF1645.5* and *OsDUF1645.6*), while Chromosomes 3, 6, 7, 11, and 12 each possessed a single gene locus. No OsDUF1645 genes were mapped to Chromosomes 4, 8, 9, or 10. The absence of OsDUF1645 members from these chromosomes may reflect lineage-specific retention patterns or evolutionary loss events during rice genome evolution. Nevertheless, although the current IRGSP annotation provides a high-quality reference, the possibility of undetected or poorly annotated loci cannot be completely excluded. Given that gene duplication is a primary driver of multigene family expansion and functional diversification, gene duplication modalities were systematically characterized using MCScanX. Following duplication, duplicated genes may undergo subfunctionalization, neofunctionalization, or differential regulatory evolution, thereby contributing to functional diversification within gene families. A total of three paralogous duplication pairs were identified within the OsDUF1645 family, encompassing both localized and genome-wide events. A single tandem duplication event was verified on Chromosome 5, involving the adjacent loci *OsDUF1645.8* and *OsDUF1645.9* (Figure 3A). In addition, two segmentally duplicated gene pairs were identified across distinct chromosomal blocks (Figure 3A). These duplicated pairs were located within conserved syntenic blocks detected by MCScanX, suggesting a possible role for large-scale chromosomal rearrangement events in the preservation and expansion of OsDUF1645 members during rice genome evolution. Genome-wide synteny analysis confirmed these findings, showing clear paths linking OsDUF1645 paralogs within conserved collinear regions (Figure 3A). Pairwise protein sequence comparison further supported the three synteny-based paralogous relationships identified within the OsDUF1645 family. The sequence identities were 62.50% for *OsDUF1645.3*/*OsDUF1645.10*, 55.88% for *OsDUF1645.6*/*OsDUF1645.11*, and 49.12% for OsDUF1645.8/OsDUF1645.10. These sequence similarities, together with the synteny evidence, provide additional support for the inferred paralogous relationships (Figure 3B). Taken together, these results indicate that both localized tandem duplication and segmental duplication events contributed to the evolutionary expansion and diversification of the OsDUF1645 gene family in rice.

### 3.5. In Silico Co-Localization and Functional Connectivity of the OsDUF1645 Family Within the Rice Abiotic Stress QTLome

**Figure 4:**
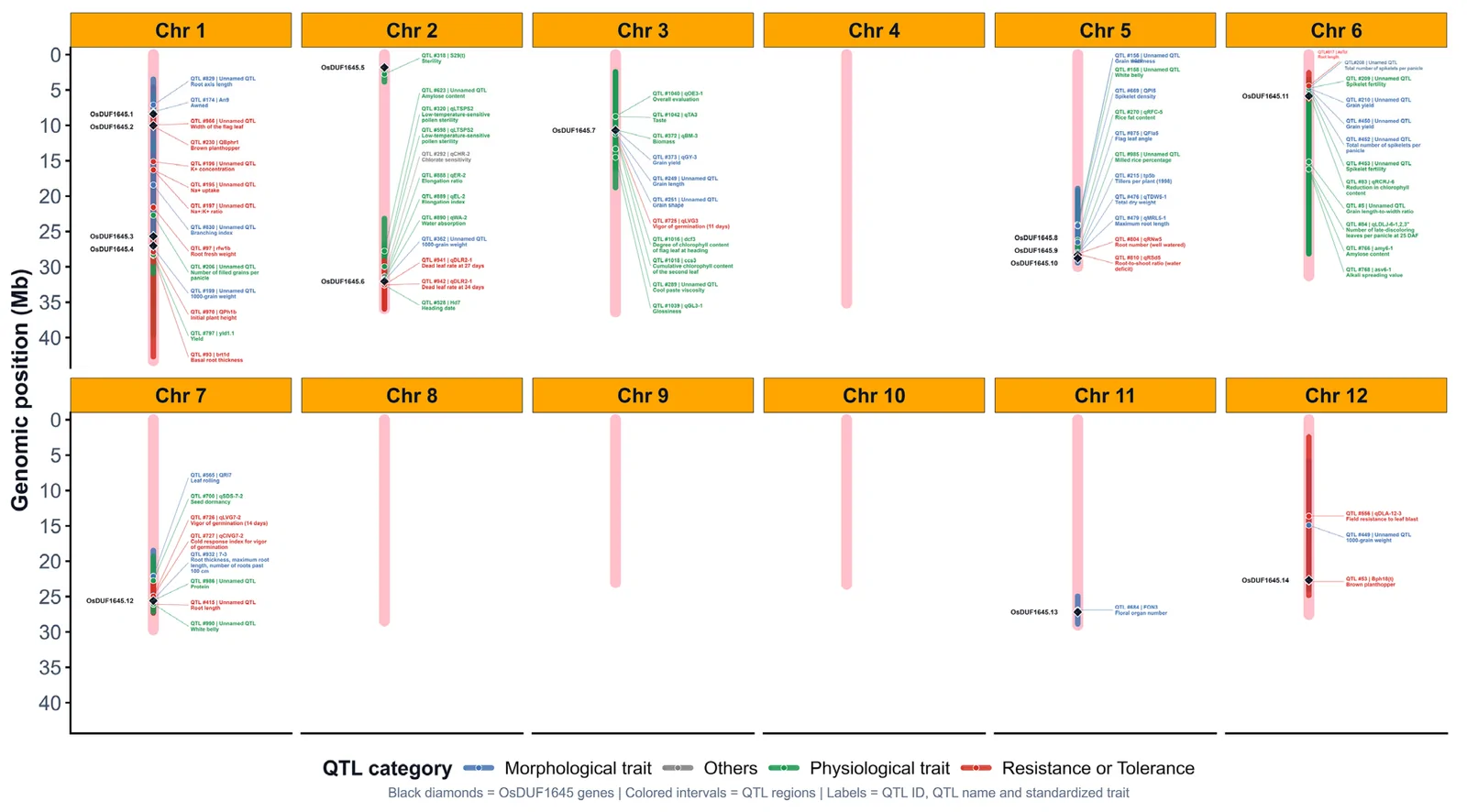
Ideogram QTL association to OsDUF1645 genes in rice chromosomes. QTL regions overlapping with the OsDUF1645 loci are displayed along the corresponding chromosomes.

**Figure 5:**
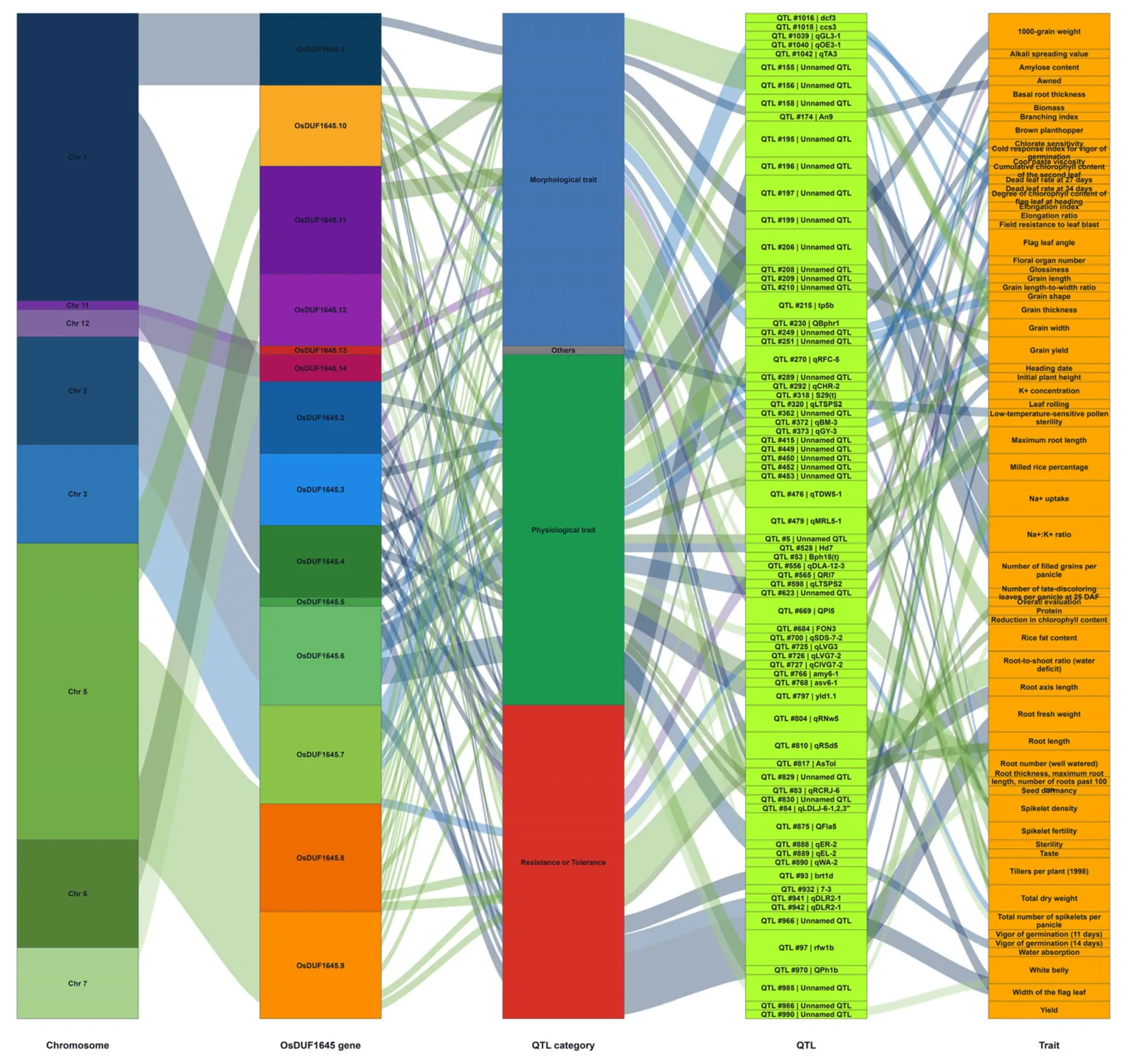
Sankey alluvial representation of the functional and regulatory relationships of the OsDUF1645 family. The plot illustrates the distribution of OsDUF1645 genes across functional, regulatory, phylogenetic, and QTL-associated categories.

To investigate the potential genetic relevance of the *OsDUF1645* family, the genomic positions of all 14 members were integrated with curated rice QTL information (Table S3). This analysis identified 112 gene–QTL associations involving 73 unique QTLs and 62 standardized traits, distributed across Chromosomes 1, 2, 3, 5, 6, 7, 11, and 12 (Table S3, Figure 4). Chromosomes 1 and 5 showed the strongest concentration of associations (Table S3, Figure 4). The Chr1 cluster (*OsDUF1645.1*–*4*) accounted for 32 associations involving 14 QTLs, including loci related to root fresh weight, Na^+^ uptake, K^+^ concentration, and Na^+^:K^+^ ratio (Table S3, Figure 4). The Chr5 cluster (*OsDUF1645.8*–*10*) accounted for 33 associations involving 12 QTLs spanning root, grain, physiological, and morphological traits (Table S3, Figure 4). *OsDUF1645.6* also showed a relatively broad association profile, with 11 QTL associations across multiple phenotypic categories (Table S3, Figure 4). An integrated alluvial network (Figure 5) showed strong multi-gene convergence on Chr1 and substantial sharing of QTL associations among the tandemly clustered Chr5 genes, particularly QTLs #215, #270, #476, #479, #669, #804, #810, #875, and #985. In contrast, *OsDUF1645.6*, *OsDUF1645.7*, *OsDUF1645.11*, and *OsDUF1645.12* showed broader and more differentiated phenotypic connectivity. These results indicated that *OsDUF1645* loci occur within genomic regions associated with diverse root, physiological, developmental, grain, yield, and stress-related traits.

### 3.6. Cis-Acting Element Analysis of OsDUF1645 Gene Promoters

To characterize the potential transcriptional regulatory diversification of the OsDUF1645 family, a standardized 1500 bp region immediately upstream of the ATG start codon was analyzed for each of the 14 OsDUF1645 genes using the PlantCARE database. This 1500 bp interval was selected to provide a consistent promoter window encompassing proximal and moderately distal regulatory regions while minimizing differences in sequence coverage among family members. Although this approach captures a substantial portion of the predicted promoter landscape, regulatory elements located farther upstream or within distal enhancers may also contribute to OsDUF1645 transcription and were not assessed in the present analysis. Across the 14 analyzed promoters, 430 CARE occurrences were assigned to three major regulatory categories, comprising 172 phytohormone-responsive occurrences (40%), 156 abiotic stress-associated occurrences (36.3%), and 94 growth- and development-associated occurrences (23.7%) (Table S4). Thus, phytohormone- and abiotic stress-related elements together accounted for approximately 76.3% of all classified CARE occurrences (Table S4). A total of 37 CARE types were identified across the OsDUF1645 promoters (Table S4). The distribution of Methyl Jasmonate (MeJA) responsive units (the core consensus CGTCA-motif and its matching TGACG-motif) shows an asymmetric topology across OsDUF1645 genes, with these motifs being heavily prioritized in candidate genes like *OsDUF1645.5*, which contains seven CGTCA-motifs and seven TGACG-motifs, but completely absent in members such as OsDUF1645.11 and *OsDUF1645.14*, indicating clear regulatory divergence (Table S4). In addition, salicylic acid (SA)-responsive elements (TCA-element, TCA motif, SARE, and as-1 motif) exhibit a specialized distribution that varies from a maximum cluster of one TCA-element and seven as-1 motifs in *OsDUF1645.5* down to a complete absence in *OsDUF1645.12* (Table S4). In rice systems, the targeted accumulation of these regulatory structures in elite loci, such as the one TCA-element and two as-1 motifs in *OsDUF1645.1* or the one TCA-element and four as-1 motifs in *OsDUF1645.8*, may correlate with known plant defense frameworks that signal downstream systemic acquired resistance (SAR) during biotic stress. Several growth- and development-associated CAREs identified in the OsDUF1645 promoters are associated with developmental and light-responsive transcriptional regulation. Their occurrence suggests that OsDUF1645 transcription may potentially integrate environmental signals with developmental programs, including light-dependent growth and photomorphogenic processes.

**Figure 6:**
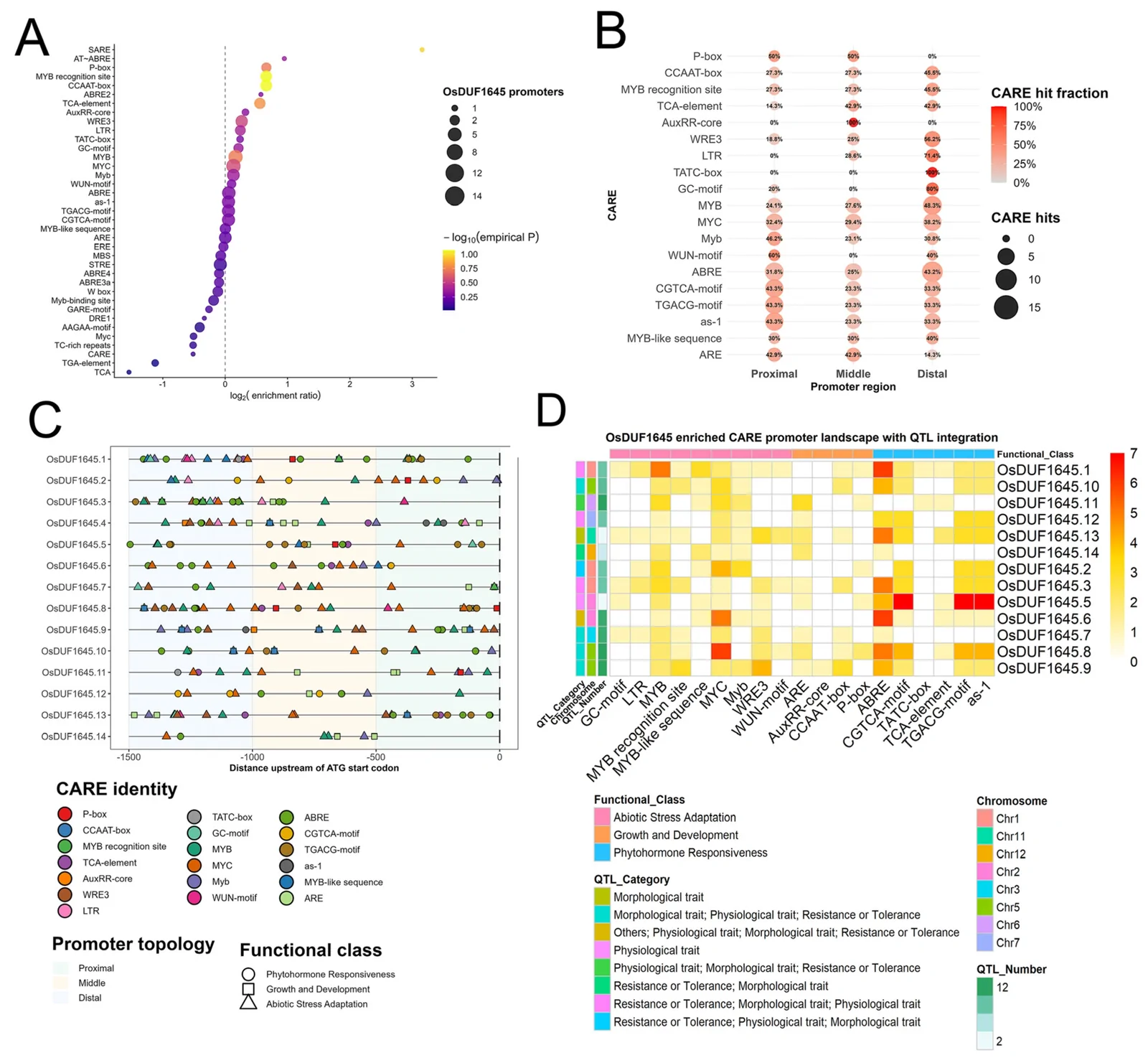
Integrated *cis*-regulatory landscape and QTL association analysis of OsDUF1645 promoter regions. (**A**) CARE enrichment relative to 1000 background rice promoters; bubble size indicates promoter frequency and color denotes functional category. (**B**) CARE regulatory topology showing conserved hubs and promoter-specific *cis*-regulatory combinations. (**C**) Spatial distribution of CAREs across 1500 bp promoters, divided into distal, middle, and proximal regions. (**D**) CARE abundance heatmap integrated with QTL annotations, showing motif profiles, chromosomal locations, QTL categories, and associated traits.

Comparison with 1000 randomly selected rice promoters revealed that several CAREs showed higher representation in the OsDUF1645 promoter set (Figure 6A). P-box showed the strongest enrichment ratio (1.58-fold), followed by CCAAT-box and MYB recognition site (1.58-fold each), TCA-element (1.47-fold), LTR (1.18-fold), and WRE3 (1.20-fold) (Figure 6A). Although these enrichment trends did not remain statistically significant after multiple-testing correction, they indicate potential regulatory features associated with the OsDUF1645 promoter architecture. Several widely distributed elements, including MYB, MYC, ABRE, ARE, CGTCA-motif, TGACG-motif, and as-1, were detected in most family members, suggesting the presence of a conserved regulatory framework shared among OsDUF1645 genes (Figure 6A). MYB and MYC elements were present in all analyzed promoters, while ABRE was detected in 12 of 14 promoters (Figure 6A), highlighting extensive potential integration of stress- and hormone-related regulatory signals. Stress-related elements, including LTR, WRE3, and MYB-associated motifs, showed increased representation among OsDUF1645 promoters compared with the genomic background (Figure 6A). Hormone-responsive elements, including ABRE, CGTCA-motif, TGACG-motif, and as-1, were also widely distributed, reflecting the potential involvement of ABA-, jasmonate-, and related signaling pathways in OsDUF1645 regulation (Figure 6A). However, the absence of significant FDR-adjusted enrichment indicates that these motifs likely represent an enhanced but not exclusive regulatory tendency of the family. 

CARE topology analysis revealed both conserved and gene-specific regulatory patterns among OsDUF1645 promoters. Frequent elements such as MYB, MYC, ABRE, CGTCA-motif, TGACG-motif, and as-1 formed regulatory hubs, whereas low-frequency elements contributed more specific promoter signatures (Figure 6B,C). CAREs were distributed across distal, middle, and proximal promoter regions, with CGTCA-motif, TGACG-motif, and as-1 enriched proximally, while WRE3 and AAGAA-motif were more distal. ABREs showed broad distribution (Figure 6B,C). These patterns indicate that OsDUF1645 regulation depends on both *cis*-element composition and spatial organization, supporting functional diversification among family members. Integration of CARE abundance and QTL information revealed substantial regulatory and genomic diversification among OsDUF1645 members (Figure 6D). Distinct promoter profiles were observed, including ABRE/MYB-rich OsDUF1645.1, CGTCA/TGACG-enriched *OsDUF1645.5*, and jasmonate-related motif enrichment in *OsDUF1645.8* and *OsDUF1645.12* (Figure 6D). These patterns corresponded with distinct QTL associations: Chromosome 1 genes were linked to root, Na^+^/K^+^, and salinity traits; Chromosome 5 genes to root development and water-deficit responses; and *OsDUF1645.11*–*12* to root architecture and cold-related traits (Figure 6D). Overall, the integrated analyses indicate a conserved yet diversified *cis*-regulatory and genetic landscape within the OsDUF1645 family. The coexistence of shared regulatory hubs and promoter-specific CARE combinations suggests that regulatory remodeling contributed to functional differentiation of OsDUF1645 family members during rice adaptation to environmental stress, developmental regulation, and agronomic trait variation. Moreover, comparison of CARE profiles with the phylogenetic classification (Figure 1) revealed both conserved and divergent regulatory features among evolutionary groups. Closely related OsDUF1645 members retained overlapping combinations of stress- and hormone-associated CAREs, whereas differences in CARE abundance and composition were also observed among members within the same phylogenetic group. This pattern could reflect that promoter architecture that may have accompanied the evolutionary diversification of the protein family.

### 3.7. In Silico Mining of Public Reference Transcriptome Networks Reveals Stress-Responsive and Regulatory Divergence Within the OsDUF1645 Family

**Figure 7:**
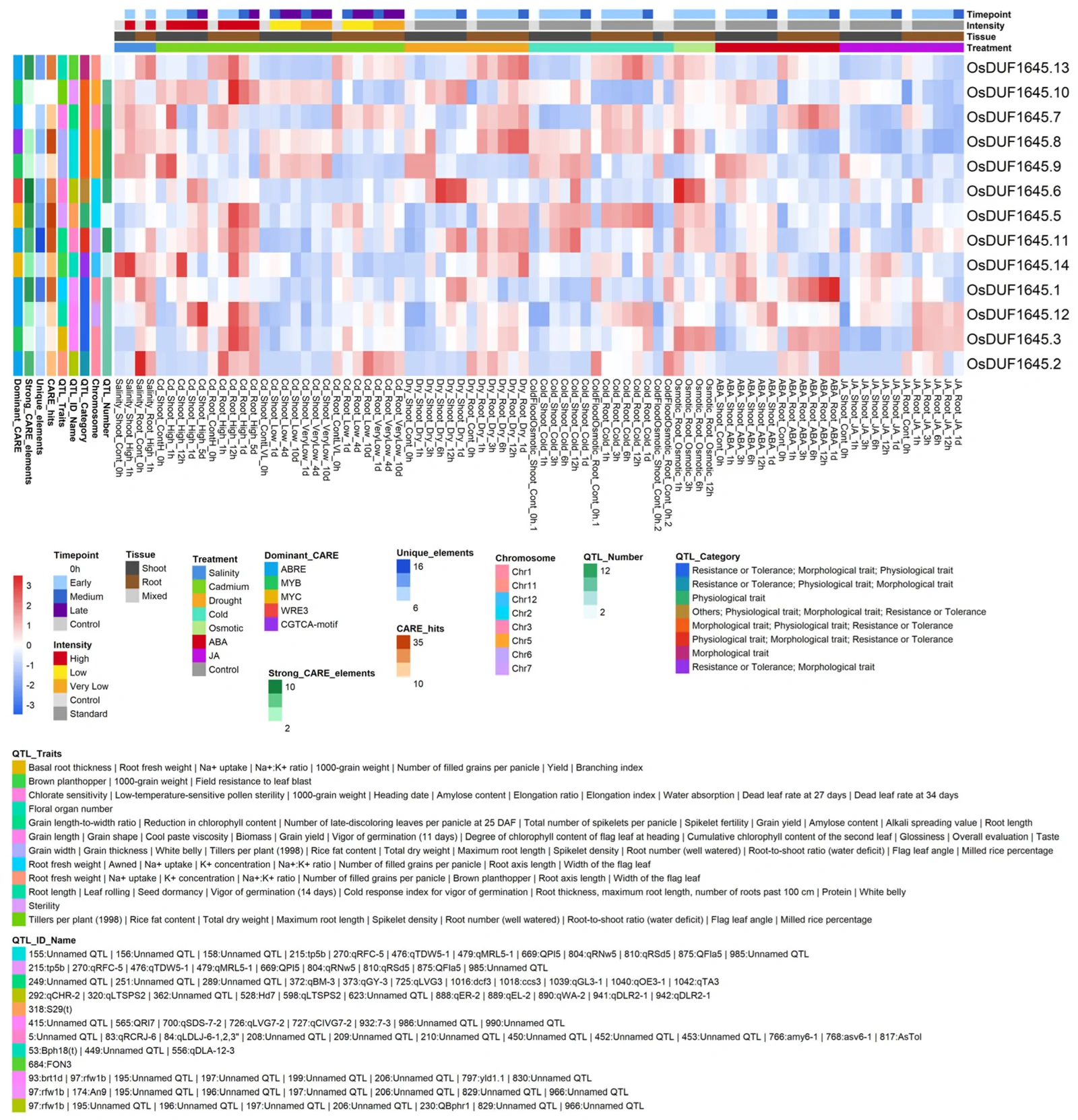
Integrated expression, *cis*-regulatory, and genetic association landscape of OsDUF1645 genes under abiotic stress and phytohormone treatments. The heatmap shows OsDUF1645 transcriptional responses to salinity, cadmium, drought, cold, osmotic stress, ABA, and JA/MeJA across tissues and time points. Color intensity represents Log_2_ fold change, with red indicating induction and blue repression. CARE annotations summarize stress- and hormone-responsive promoter elements, while QTL annotations indicate associated genomic regions and traits, including ion homeostasis, root architecture, yield, and stress tolerance. Integration of expression, CARE, and QTL profiles highlights functional diversification and prioritizes OsDUF1645 genes with broad or condition-specific stress responses and supporting regulatory and genetic evidence.

The in silico expression analysis based on publicly available rice transcriptome datasets (TENOR framework) revealed the expression profiles of OsDUF1645 family members across multiple abiotic stress and hormone-associated conditions (Figure 7, Tables S5 and S6). The integrated analysis considered both induction (Log2FC > 1) and repression (Log2FC < −1) events across stress treatments, tissues, and temporal phases, revealing that OsDUF1645 genes display distinct regulatory strategies ranging from broad stress responsiveness to condition-specific transcriptional modulation. Among the family members, *OsDUF1645.3*, *OsDUF1645.5*, *OsDUF1645.8*, *OsDUF1645.10*, *OsDUF1645.11*, and *OsDUF1645.12* showed the broadest transcriptional responses, responding to multiple environmental and hormonal treatments including cadmium, drought, cold, osmotic stress, ABA, and JA. These genes displayed high numbers of transcriptional response events, with *OsDUF1645.3*, *OsDUF1645.10*, and *OsDUF1645.11* showing particularly strong activation profiles, characterized by high positive Log2FC values and limited repression frequency. In contrast, *OsDUF1645.8*, *OsDUF1645.7*, *OsDUF1645.9*, and *OsDUF1645.13* exhibited substantial transcriptional plasticity, with frequent downregulation events under multiple stress conditions, indicating complex regulatory control rather than simple stress-induced activation. Notably, the transcriptional profiles of the Chromosome 5 OsDUF1645 members further illustrated regulatory divergence among closely linked genes. The tandemly duplicated *OsDUF1645.8* and *OsDUF1645.9* displayed markedly different stress-responsive patterns despite their adjacent genomic positions. *OsDUF1645.8* responded to five stress conditions, including salinity, cadmium, drought, cold, and osmotic stress, with 12 early-response events, a maximum Log2FC of 2.615, and a mean Log2FC of 1.627. In contrast, OsDUF1645.9 responded to only cold and osmotic stress, with two early-response events and a lower maximum Log2FC of 1.197. Furthermore, OsDUF1645.9 showed 12 strong repression events, whereas no strong repression events were detected for *OsDUF1645.8*. These contrasting transcriptional profiles point to possible substantial post-duplication regulatory divergence between the tandem duplicates, which could underlie their functional specialization under environmental stress, although this possibility requires further functional validation. 

Integration of expression kinetics with promoter and QTL information identified *OsDUF1645.8* as the highest-ranked candidate, combining multi-stress responsiveness, strong promoter regulatory architecture, and extensive QTL associations (Figure 7, Table S6). Despite its strong positive induction profile, *OsDUF1645.8* also displayed frequent repression events under several stress and hormone treatments, suggesting dynamic transcriptional regulation depending on stress intensity, tissue context, or treatment duration (Figure 7, Table S6). Similarly, *OsDUF1645.3* exhibited a strong integrated candidate score due to broad stress responsiveness, high expression amplitude, strong promoter support, and moderate QTL association, with relatively limited repression compared with other family members (Figure 7, Table S6). Temporal analysis revealed clear differences between rapid and sustained transcriptional responses. Several genes, including *OsDUF1645.3*, *OsDUF1645.5*, *OsDUF1645.10*, *OsDUF1645.11*, and *OsDUF1645.12*, displayed early transcriptional activation during short-term stress exposure (3–12 h), followed by continued responses during prolonged treatments (1–10 days). In contrast, genes such as *OsDUF1645.9* and *OsDUF1645.13* exhibited extensive repression across multiple treatments, including cadmium, drought, cold, osmotic, ABA, and JA conditions, indicating that certain family members may function through stress-associated transcriptional attenuation or pathway-specific restriction (Figure 7, Table S6). Cadmium treatment activated several members, including *OsDUF1645.3*, *OsDUF1645.5*, *OsDUF1645.8*, *OsDUF1645.10*, *OsDUF1645.11*, and *OsDUF1645.12*, while simultaneously causing repression of other loci such as *OsDUF1645.9* and *OsDUF1645.13* (Figure 7,Table S5). This divergent response suggests functional specialization within the family. ABA- and JA-responsive regulation showed substantial overlap across the OsDUF1645 family (Figure 7, Table S5). Ten of the 13 OsDUF1645 members exhibited positive responses to ABA, while nine responded positively to JA, with nine genes showing responsiveness to both hormones (Figure 7, Table S5). Several members, particularly *OsDUF1645.3*, *OsDUF1645.5*, and *OsDUF1645.11*, displayed consistently positive and relatively strong transcriptional responses under both ABA and JA treatments (Figure 7, Table S5), pointing to their potential involvement in a common hormone-responsive regulatory program. 

The extensive overlap between ABA- and JA-responsive members is consistent with potential integration of ABA- and JA-mediated signaling during abiotic stress adaptation. However, because the present analysis is based on transcriptional co-responsiveness rather than hormone perturbation or pathway-interference experiments, it does not establish direct ABA–JA dependency or causal cross-talk. Root-associated expression responses were observed for multiple OsDUF1645 members, including genes showing induction in roots such as *OsDUF1645.3*, *OsDUF1645.10*, *OsDUF1645.5*, and *OsDUF1645.8*. Notably, these responses coincide with QTL associations involving root-related traits, including root length, root number, root fresh weight, root thickness, and root-to-shoot ratio, suggesting a potential link between OsDUF1645 regulation and root developmental plasticity under environmental stress. When combined with promoter *cis*-element enrichment and QTL localization, these expression patterns identify *OsDUF1645.8*, *OsDUF1645.3*, *OsDUF1645.11*, *OsDUF1645.6*, and *OsDUF1645.10* as the strongest candidates for future functional validation in abiotic stress adaptation, while highlighting additional members that may participate through stress-dependent transcriptional repression mechanisms.

### 3.8. qRT-PCR Validation of OsDUF1645 Gene Expression Under Different Abiotic Stresses

**Figure 8:**
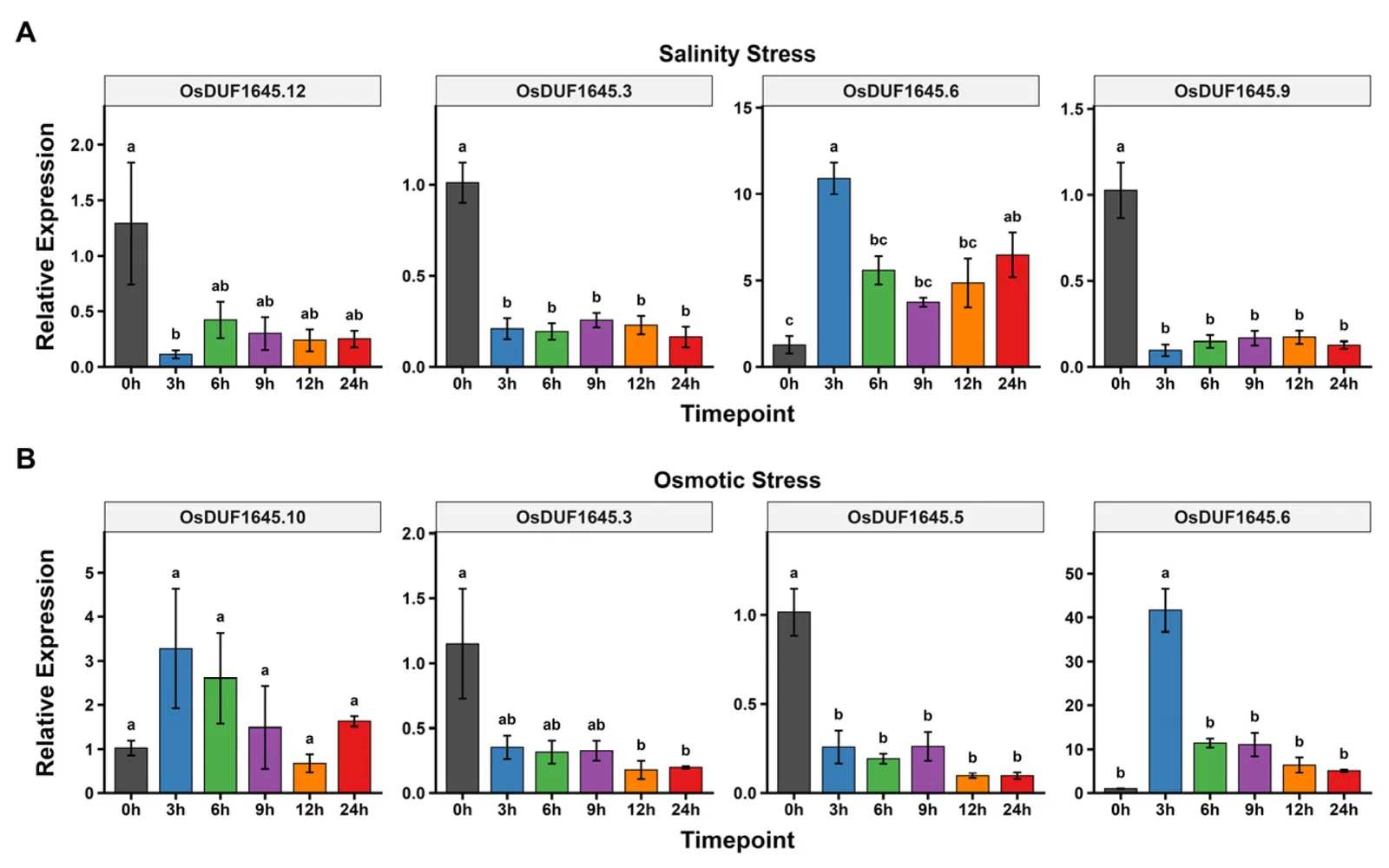
qRT-PCR validation of OsDUF1645 family gene expression under PEG6000 and NaCl treatments. The relative expression levels of OsDUF1645 genes were analyzed under salt (NaCl) (**A**) and PEG6000 (**B**) treatments at different time points (0, 3, 6, 9, 12, and 24 h). Expression levels were normalized to the internal reference gene *OsActin* and calculated using the 2^−ΔΔCt^ method. Data are represented as the mean ±SEM of three biological replicates (*n* = 3), error bars are on corresponding group bars in the plot. Different lowercase letters indicate significant differences among timepoints, whereas the same letters indicate no significant difference, based on one-way ANOVA followed by Tukey’s post hoc test (*p* < 0.05).

Six OsDUF1645 members (*OsDUF1645.3*, *OsDUF1645.5*, *OsDUF1645.6*, *OsDUF1645.9*, *OsDUF1645.10*, and *OsDUF1645.12*) were subjected for RT-qPCR validation across a 24 h time course under salinity (NaCl) and osmotic (PEG6000) stress treatments (Figure 8). These genes were selected to represent distinct regulatory response profiles identified through the integrated analysis. *OsDUF1645.3*, *OsDUF1645.5*, and *OsDUF1645.10* were prioritized because of their strong and broad stress-responsive induction, whereas *OsDUF1645.12* represented a broadly responsive gene with comparatively moderate induction. *OsDUF1645.6* was included as an intermediate-response candidate with substantial QTL support, while *OsDUF1645.9* was selected as a contrasting condition-responsive member characterized by a relatively high proportion of repression events and strong negative responses. The experimental validation confirmed that individual members of the OsDUF1645 family exhibit highly specific and distinct transcriptional profiles in response to both environmental cues. Under salinity stress, *OsDUF1645.6* was rapidly and strongly induced, reaching its maximum expression amplitude at 3 h post-treatment before undergoing a gradual decline (Figure 8A). Conversely, *OsDUF1645.9*, *OsDUF1645.12*, and *OsDUF1645.3* were markedly repressed by salinity (Figure 8A), exhibiting a sharp drop in transcript levels within 3 h and maintaining low levels throughout the remainder of the treatment period. The repression observed for *OsDUF1645.5*, *OsDUF1645.9*, and *OsDUF1645.12* may indicate stress-dependent transcriptional prioritization, tissue-specific regulation, or selective attenuation of pathways that are not required during particular stress phases. The reduced transcript accumulation may not necessarily reflect impaired stress tolerance, but could instead be an alternative regulatory strategy within the OsDUF1645 family. 

Under PEG6000-induced osmotic stress, *OsDUF1645.6* exhibited a similar early-response profile, significantly upregulating at 3 h, with approximately 3.2-fold induction (log2FC = 1.68) at 3 h, before decreasing incrementally (Figure 8B). The rapid activation of OsDUF1645.6 is consistent with early salinity signaling events involving osmotic perception, ion homeostasis regulation, calcium-dependent signaling, and ABA-associated stress responses, which commonly activate downstream protective transcriptional programs. In contrast, *OsDUF1645.10* showed no significant change under PEG6000 treatment (Figure 8B). Meanwhile, *OsDUF1645.5* and *OsDUF1645.3* were uniformly downregulated across almost all examined time points under osmotic stress (Figure 8B). Importantly, the transcriptional shifts detected by qRT-PCR were generally consistent with the trends observed in public RNA-seq datasets. Quantitative comparison using log2-transformed expression values showed moderate-to-strong concordance for most validated OsDUF1645 genes, including *OsDUF1645.10* (R^2^ = 0.40), *OsDUF1645.5* (R^2^ = 0.36), and *OsDUF1645.6* (R^2^ = 0.72). These results support the reliability and biological relevance of the transcriptome-based predictions. Importantly, the transcriptional shifts captured by qRT-PCR were generally consistent with trends observed in public RNA-seq datasets, demonstrating the probable reliability and biological relevance of the preliminary transcriptome-wide analysis.

**Figure 9:**
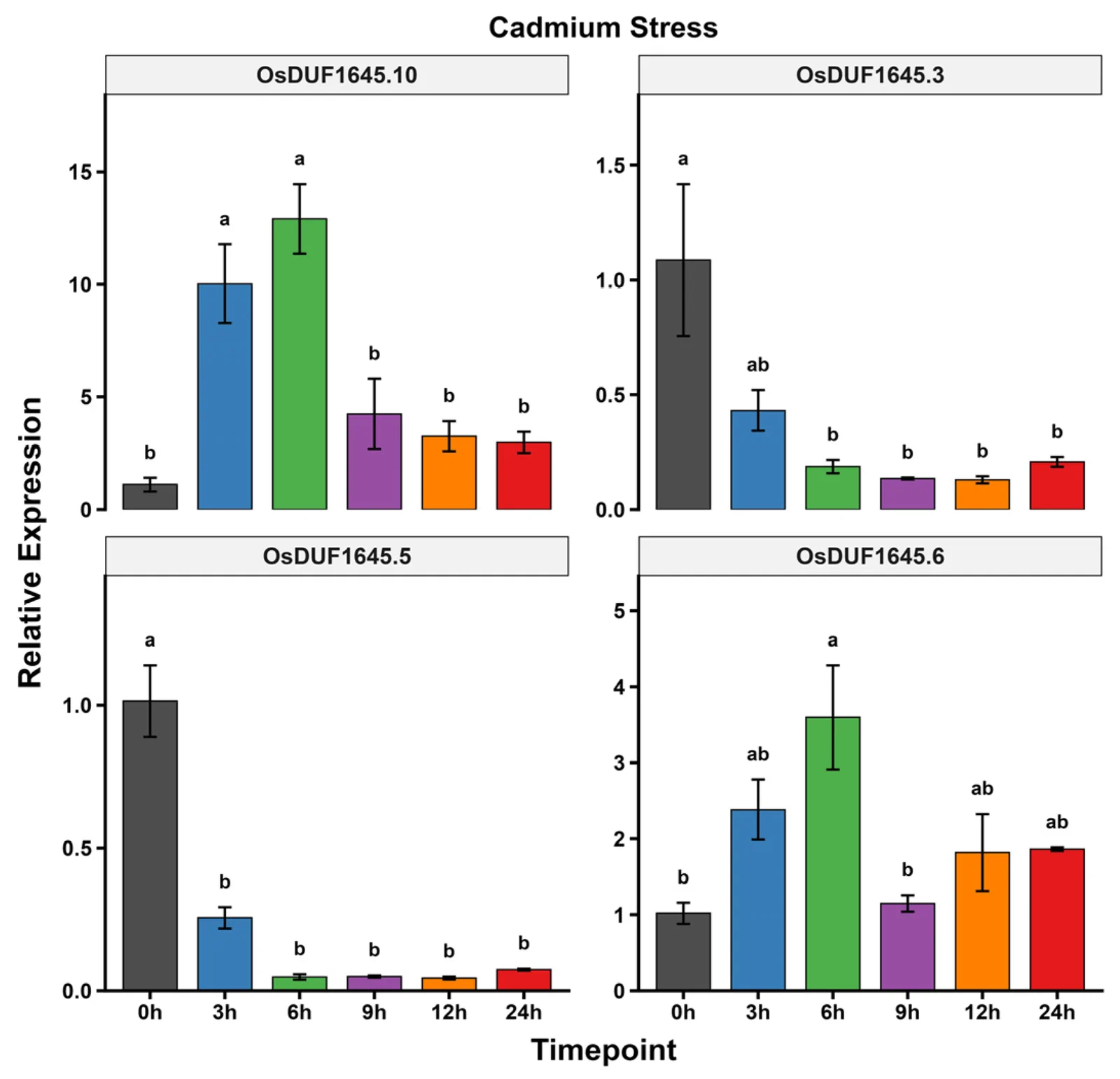
Expression profiles of four OsDUF1645 genes under CdCl_2_ stress. The relative expression levels of four OsDUF1645 genes were analyzed under CdCl_2_ treatments at different time points (0, 3, 6, 9, 12, and 24 h) by qRT-PCR. Expression levels were normalized to the internal reference gene *OsActin* and calculated using the 2^−ΔΔCt^ method. Data are represented as the mean ± SEM of three biological replicates. Different lowercase letters indicate significant differences among timepoints, whereas the same letters indicate no significant difference, based on one-way ANOVA followed by Tukey’s post hoc test (*p* < 0.05).

Given that the exploratory transcriptome-wide analysis indicated responsiveness to heavy-metal toxicity among specific family members, four representative OsDUF1645 genes were selected to validate their transcriptional profiles under cadmium (Cd^2+^) stress using qRT-PCR (Figure 9). The experimental results demonstrated that these candidate genes exhibit highly distinct temporal expression kinetics following heavy metal exposure. Specifically, *OsDUF1645.10* was induced during the early phases of cadmium stress; its transcript abundance surged markedly at 3 h, achieved a maximal peak at 6 h post-treatment, and subsequently underwent a gradual decline from 9 to 24 h, though maintaining an elevated baseline relative to the untreated control (0 h). In sharp contrast, *OsDUF1645.3* displayed a distinct downward trajectory, with transcript levels dropping precipitously within 3 h of cadmium exposure and remaining suppressed for the remainder of the experimental period. Concurrently, *OsDUF1645.6* was also triggered by heavy metal stress, showing significant upregulation at 3 h, peaking at 6 h, and then declining steadily at subsequent time points. Notably, *OsDUF1645.10* and *OsDUF1645.6* exhibited highly synchronized temporal expression waves under cadmium toxicity. Both genes were actively recruited during early-stage heavy-metal signaling cascades and reached their maximum transcript levels concurrently at 6 h, suggesting potential functional synergy or shared upstream regulatory networks in response to heavy-metal stress in rice.

## 4. Discussion 

Although DUFs were originally defined on the basis of limited functional annotation, increasing evidence indicates that many DUF-containing proteins represent biologically active and evolutionarily conserved components involved in plant growth, development, hormone regulation, and environmental adaptation [5,35]. For example, functional characterization of a DUF246 family glycosyltransferase-like protein demonstrated its essential role in pectic arabinogalactan biosynthesis and male fertility, providing direct evidence that DUF-containing proteins can participate in fundamental developmental processes, rather than representing nonfunctional protein regions [5,35]. Furthermore, evolutionary analyses of DUF26-containing proteins revealed that DUF domains have undergone lineage-specific diversification and functional specialization, contributing to plant proteins associated with extracellular signaling and stress-related responses [36]. Recent functional and genome-wide studies of additional DUF families, including DUF568, DUF668, DUF724, DUF868, and DUF247, have further demonstrated their involvement in developmental regulation, hormone responses, and abiotic stress adaptation [37,38,39,40,41]. Recent genome-wide analyses of different DUF families, including DUF4228, DUF1216, DUF568, and DUF668, have consistently shown that integrated characterization of phylogeny, gene structure, conserved motifs, promoter composition, and expression profiles provides valuable evidence for predicting the potential functions of previously uncharacterized genes [21,38,41,42,43]. In particular, promoter analysis provides insights into the potential regulatory mechanisms underlying DUF gene activation by identifying *cis*-regulatory elements associated with phytohormones, stress responses, and developmental signals, whereas expression profiling across tissues and environmental conditions helps establish associations between specific DUF members and biological processes. In rice, such integrative approaches have successfully revealed that DUF genes exhibit tissue-preferential expression patterns and dynamic transcriptional responses to abiotic stresses, supporting their predicted roles in plant development and environmental adaptation [40,41]. Similarly, analyses of OsDUF568 and TaDUF724 families demonstrated that hormone- and stress-responsive promoter elements correspond with experimentally observed transcriptional changes under abiotic and hormonal treatments, highlighting the importance of combining regulatory landscape analysis with expression data for functional inference [38,39]. In this context, the present study establishes an integrative framework for the characterization of the OsDUF1645 family in rice by combining phylogenetic analysis, gene structure and conserved motif identification, promoter *cis*-element profiling, stress-responsive expression analysis, and co-localization with validated stress-related QTLs. This multi-dimensional approach provides insights into the evolutionary relationships, regulatory architecture, expression dynamics, and genetic associations of OsDUF1645 members, thereby prioritizing candidate genes and generating testable hypotheses for future functional validation. Our screening identified 14 non-redundant OsDUF1645 transcripts, although this number does not represent identical membership revealed by Cui, Wang, Zhou, Li, Huang, Yin, Zhao, Lin, Xia and Xu [27]. Locus-by-locus comparison revealed three loci absent from our current set (*LOC_Os01g39760*, *LOC_Os05g06340*, and *LOC_Os05g09590*) and two newly identified members (*LOC_Os01g17420* and *LOC_Os02g04130*). These differences likely reflect updates in genome annotation and domain-identification criteria, highlighting that OsDUF1645 family membership may vary across annotation versions and identification pipelines. The integrated QTL, expression, and genomic analyses indicate that the OsDUF1645 family has undergone substantial functional diversification, with distinct genomic branches associated with specific stress-adaptive traits. A prominent example is the Chromosome 5 tandem cluster (*OsDUF1645.8*–*10*), which overlaps root-related QTLs for root number and root-to-shoot biomass under water deficit. Their coordinated root-associated stress responses support potential specialization in root development and drought adaptation. In contrast, *OsDUF1645.12* on Chromosome 7 overlaps cold- and root-related QTLs and responds to cadmium, cold, drought, and JA, suggesting broader integration of environmental and hormonal signals. The Chromosome 1 cluster (*OsDUF1645.1*–*3*) overlaps validated QTLs associated with Na^+^ uptake, K^+^ concentration, and Na^+^/K^+^ homeostasis, while its rapid shoot induction under salinity supports a potential role in ion-stress responses. Notably, *OsDUF1645.6* on Chromosome 2 overlaps the soil-stress QTL qDLR2-1 and showed strong, persistent induction across ABA, JA, salinity, drought, and cadmium treatments, further supported by qRT-PCR validation. Collectively, these findings position distinct OsDUF1645 members as candidates linking stress-responsive transcription, promoter regulation, and agronomic QTLs, providing a framework for functional validation and future breeding-oriented studies.

The presence of DUF1645 genes in rice, maize, and Arabidopsis suggests that this family is evolutionarily conserved in both monocot and dicot plants. However, phylogenetic analysis showed that maize and rice DUF1645 members were divided into three groups, whereas Arabidopsis members were distributed into only two corresponding groups. The observed difference in phylogenetic group distribution may reflect lineage-specific evolutionary divergence, but it could also be influenced by the smaller number of Arabidopsis sequences included in the analysis; therefore, broader taxonomic sampling will be required to distinguish these possibilities. Similar evolutionary patterns have been reported in other plant gene families, in which lineage-specific clustering reflects differential expansion, retention, and potential functional divergence among subgroups [36,43,44]. For example, genome-wide analyses of the DUF family in rice have identified DUF-containing genes among candidate loci associated with submergence tolerance, alongside established stress-responsive families such as WRKY, F-box, zinc-finger, and protein kinase genes [45]. Similarly, transcriptomic analyses of salt-tolerant Zoysia japonica identified DUF genes together with WRKY and bHLH transcription factors as components of the salt-stress response [25]. Comparative evolutionary analyses of other stress-associated plant gene families, including zinc finger-BED proteins, have also demonstrated lineage-specific diversification, gene gain/loss, and structural divergence across land plants [46]. These observations suggest that the evolutionary diversification of DUF1645 may likewise reflect lineage-specific retention and expansion associated with adaptive processes, although the present phylogenetic analysis alone does not establish a direct functional relationship between DUF1645 expansion and stress-responsive transcription factor families such as NAC or WRKY. Notably, recent rice GWAS has also identified multiple DUF-containing genes, together with WRKY, ERF, and MYB transcription factors, as candidate loci associated with biomass-related traits [47], further supporting the broader relevance of DUF diversification to agriculturally important rice phenotypes.

Gene duplication is widely recognized as an important force driving the expansion and functional divergence of plant gene families, mainly through segmental, tandem, and whole-genome duplication [44,48,49,50]. In the present study, both tandem and segmental duplication events were identified in the rice DUF1645 family, suggesting that these duplication modes contributed to the expansion and retention of OsDUF1645 genes in the rice genome. Gene structure analysis provided additional support for this interpretation. Moreover, all OsDUF1645 genes displayed a conserved exon–intron organization, with all members lacking introns. Such conserved intronless architecture may facilitate rapid transcriptional responses under stress conditions, as genes with few or no introns are often associated with fast regulatory responses in plants [51,52]. Members within the same subgroup also shared similar motif compositions, whereas differences were observed among subgroups, suggesting possible functional divergence during evolution. Consistent with this hypothesis, our integrated expression analysis revealed that motif conservation among OsDUF1645 subgroups was accompanied by both shared and divergent stress-responsive transcriptional patterns. While several members within the family exhibited common responses to multiple abiotic stresses, including salinity, cadmium, drought, cold, osmotic stress, and hormone treatments, individual genes showed distinct levels of induction, repression, and stress specificity. For instance, *OsDUF1645.8* and *OsDUF1645.6* exhibited broad multi-stress responsiveness with strong promoter regulatory support, but differed in their relative activation/repression profiles, whereas *OsDUF1645.3* and *OsDUF1645.10* displayed strong induction across multiple conditions despite differences in QTL association and promoter composition. These results suggest that conserved motifs may maintain essential structural features of OsDUF1645 proteins, whereas subgroup-specific motif divergence, together with variation in promoter *cis*-regulatory landscapes, may contribute to functional specialization and differential stress adaptation among family members. The promoter composition and expression profiles of OsDUF1645 genes provide additional clues to their potential biological roles [53,54]. Multiple *cis*-acting elements associated with phytohormone responsiveness and abiotic stress, including ABRE, TGACG motif, MBS, LTR, and G-box, were identified in the promoters of OsDUF1645 genes, suggesting that they may help integrate developmental, hormonal, and environmental signals. 

The expansion and structural diversification of DUF families in plants are frequently linked to evolutionary adaptation under fluctuating environmental pressures. 

Accumulating evidence indicates that DUF-containing proteins participate in plant responses to both abiotic and biotic stresses [4,27,55] supporting the possibility that OsDUF1645 genes are also involved in stress adaptation. Importantly, our integrated promoter, transcriptomic, and QTL analyses suggest that OsDUF1645 genes may function within established stress-responsive regulatory networks. Stress- and hormone-responsive *cis*-elements and coordinated expression under ABA, JA, salinity, drought, cold, osmotic, and heavy-metal stresses support potential links to major stress-signaling pathways. Notably, *OsDUF1645.6* showed broad multi-stress activation, while the Chromosome 1 cluster within QTL regions associated with Na^+^/K^+^ homeostasis displayed early induction. Together, these findings suggest that OsDUF1645 members may contribute to the regulation of rice adaptation to diverse environmental stresses. Consistent with this, several members responded markedly to ABA and JA treatments, but the magnitude and direction of regulation varied substantially among family members. *OsDUF1645.3*, *OsDUF1645.5*, and *OsDUF1645.11* showed consistent transcriptional activation. OsDUF1645.9 displayed a mixed regulatory pattern, whereas *OsDUF1645.13* was mainly repressed. These differences indicate that OsDUF1645 members are differentially integrated into ABA- and JA-mediated signaling networks, with some members acting as hormone-responsive activators and others showing stress-associated transcriptional suppression, suggesting functional specialization within this family. The differential promoter composition and expression responses observed among OsDUF1645 members suggest that these genes may have undergone regulatory divergence during evolution and may therefore function in different developmental or stress-related contexts in rice. However, because transcriptional responsiveness alone does not establish physiological function, these results should be regarded as useful clues for functional prediction and candidate gene selection.

Previous functional studies of DUF1645 family members support this interpretation. *OsSGL*, a DUF1645 domain-containing protein from rice, has been reported to enhance drought tolerance and improve grain length and yield [27,56], suggesting that DUF1645 genes may function in both stress adaptation and the regulation of agronomic traits. In addition, Poaceae orthologs of *OsSGL* positively regulate drought tolerance, grain length, and grain weight in rice [28], suggesting partial functional conservation of DUF1645 homologs in grasses. *OsSGL* was also shown to regulate starch biosynthesis and grain quality, further expanding the functional significance of DUF1645 genes in rice [57]. Therefore, combined with our phylogenetic, duplication, exon–intron structure, promoter, QTLome co-localization and expression analyses, these findings support the view that the DUF1645 family is structurally conserved but functionally diversified, and that some members may act at the interface between abiotic stress adaptation and developmental regulation.

Our study presents some limitations. First, this study integrates computational, transcriptomic, QTL, promoter, and qRT-PCR evidence; however, these associations do not establish causality. Functional validation using gene editing, knockdown, or overexpression is therefore required to confirm the roles of candidate OsDUF1645 genes. Second, qRT-PCR validation covered only six family members and used a single reference gene, so broader validation with additional genes and reference controls would strengthen the findings. Finally, CARE analysis was limited to the proximal 1.5 kb promoter region and may not capture distal regulatory elements or chromatin effects. Promoter-reporter assays and ATAC-seq could further define the regulatory mechanisms underlying OsDUF1645 expression.

## 5. Conclusions

In this study, we identified and characterized 14 non-redundant OsDUF1645 genes with a highly conserved intronless architecture and integrated their genomic positions, promoter features, expression profiles, and QTL associations to investigate their potential roles in rice stress adaptation. Mapping against the validated rice stress QTLome revealed 112 physical co-localization events, indicating that distinct OsDUF1645 members are located within genomic regions associated with important agronomic traits. Functional diversification was supported by the distinct genomic and transcriptional patterns observed among family members, including the Chromosome 1 cluster (*OsDUF1645.1*, *OsDUF1645.2*, *OsDUF1645.3*, and *OsDUF1645.4*) associated with salinity-related QTLs, the Chromosome 5 tandem cluster (*OsDUF1645.8*, *OsDUF1645.9*, and *OsDUF1645.10*) associated with root and water-deficit traits, and *OsDUF1645.6* as a highly responsive candidate under multiple abiotic and hormonal stresses. Promoter analysis and stress-responsive expression profiling further suggest that *OsDUF1645.6* genes are integrated into broader abiotic stress regulatory networks. The presence of ABA-, JA-, drought-, salinity-, and low-temperature-responsive *cis*-elements, together with differential transcriptional responses under stress conditions, indicates that individual members may function as distinct modulators within hormone-mediated and environmental adaptation pathways. However, the current findings remain predictive, and functional validation through genetic manipulation and biochemical approaches will be required to determine their precise physiological roles. Overall, this multi-omics framework provides a systematic approach for prioritizing OsDUF1645 candidates and establishes a foundation for future functional studies and breeding strategies aimed at improving stress resilience in rice.

## Figures and Tables

**Table 1:** Basic characteristics of the OsDUF1645 genes identified in *O. sativa*.

Gene Name	Accession Number		Protein		
	RAP_ID	MSU_ID	Size (aa)	MW a (Da)	pI b
*OsDUF1645.1*	Os01g0253500	LOC_Os01g14970	318	32,493.2	8.59
*OsDUF1645.2*	Os01g0281301	LOC_Os01g17420	247	25,337.3	11.62
*OsDUF1645.3*	Os01g0639600	LOC_Os01g45250	248	26,724.2	8.5
*OsDUF1645.4*	None	LOC_Os01g47270	264	28,519.1	5.48
*OsDUF1645.5*	Os02g0134200	LOC_Os02g04130	255	26,725.4	9.8
*OsDUF1645.6*	Os02g0761300	LOC_Os02g52380	430	44,585.5	9.11
*OsDUF1645.7*	Os03g0303100	LOC_Os03g19090	356	37,143.5	10.56
*OsDUF1645.8*	Os05g0568600	LOC_Os05g49350	231	24,054.4	6.28
*OsDUF1645.9*	Os05g0568800	LOC_Os05g49370	254	27,067.4	10.13
*OsDUF1645.10*	Os05g0578100	LOC_Os05g50230	306	31,932.7	5.7
*OsDUF1645.11*	Os06g0215100	LOC_Os06g11150	415	43,892.4	10.18
*OsDUF1645.12*	Os07g0619500	LOC_Os07g42740	329	34,277.6	11.22
*OsDUF1645.13*	Os11g0672900	LOC_Os11g44920	340	35,668.9	10.7
*OsDUF1645.14*	Os12g0556900	None	345	36,289.7	11.65

Note: ^a^ Molecular weight of the amino acid sequence; ^b^ isoelectric point.

## Data Availability

The original contributions presented in this study are included in the article and its Supplementary Materials. Further inquiries can be directed to the corresponding author.

## Supplementary Materials

The following supporting information can be downloaded at: https://www.mdpi.com/article/10.3390/genes17091044/s1, Table S1: List of OsDUF1645 identified in this study; Table S2: List of primers used in this study; Table S3: List of QTLs associated with OsDUF1645 genes; Table S4: List of *cis*-acting regulatory elements (CAREs) associated with OsDUF1645 genes; Table S5. Expression profile of OsDUF1645 genes based on TENOR data; Table S6. Integrated CARE, QTL, and expression data of OsDUF1645 genes.
